# Nonnutritive sweeteners can promote the dissemination of antibiotic resistance through conjugative gene transfer

**DOI:** 10.1038/s41396-021-00909-x

**Published:** 2021-02-15

**Authors:** Zhigang Yu, Yue Wang, Ji Lu, Philip L. Bond, Jianhua Guo

**Affiliations:** Advanced Water Management Centre, The University of Queensland, St. Lucia, Brisbane, QLD Australia

**Keywords:** Microbial ecology, Water microbiology

## Abstract

Antimicrobial resistance (AMR) poses a worldwide threat to human health and biosecurity. The spread of antibiotic resistance genes (ARGs) via conjugative plasmid transfer is a major contributor to the evolution of this resistance. Although permitted as safe food additives, compounds such as saccharine, sucralose, aspartame, and acesulfame potassium that are commonly used as nonnutritive sweeteners have recently been associated with shifts in the gut microbiota similar to those caused by antibiotics. As antibiotics can promote the spread of antibiotic resistance genes (ARGs), we hypothesize that these nonnutritive sweeteners could have a similar effect. Here, we demonstrate for the first time that saccharine, sucralose, aspartame, and acesulfame potassium could promote plasmid-mediated conjugative transfer in three established conjugation models between the same and different phylogenetic strains. The real-time dynamic conjugation process was visualized at the single-cell level. Bacteria exposed to the tested compounds exhibited increased reactive oxygen species (ROS) production, the SOS response, and gene transfer. In addition, cell membrane permeability increased in both parental bacteria under exposure to the tested compounds. The expression of genes involved in ROS detoxification, the SOS response, and cell membrane permeability was significantly upregulated under sweetener treatment. In conclusion, exposure to nonnutritive sweeteners enhances conjugation in bacteria. Our findings provide insight into AMR spread and indicate the potential risk associated with the presence of nonnutritive sweeteners.

## Introduction

Antimicrobial resistance (AMR) is recognized as one of the greatest health threats that human beings face now and in the coming decades [1–3]. Globally, 700,000 people die due to infections caused by resistant bacteria every year. It is estimated that 10 million people will be killed by infections due to AMR by 2050 if immediate action is not taken now [4]. The emergence and propagation of antibiotic resistance genes (ARGs) that confer AMR to bacteria is generally attributed to the misuse or overuse of antibiotics [5–7]. The spread of ARGs among different bacterial species is mainly driven by horizontal gene transfer (HGT). Conjugation is a significant HGT mechanism, disseminating ARGs by transferring mobile genetic elements (MGEs), including plasmids, integrons, and transposons [8–10]. ARGs carried on MGEs can be transferred by cell-to-cell contact through a pilus or pore channel connecting the host and recipient bacteria [11]. This process may occur frequently within genera but also at lower frequency across genera [12, 13].

Nonnutritive sweeteners, approved by the U.S. Food and Drug Administration (FDA), are widely being introduced as safe sugar substitutes in food and beverages as they provide a sweet taste but low calories or energy. The consumption of nonnutritive sweeteners has increased because of their potential to improve the health of individuals suffering from obesity, glucose intolerance, type II diabetes mellitus, and metabolic derangements [14–16]. The global consumption of these compounds is estimated to be approximately 117,000 metric tons annually [17]. After intake of food or beverages, these compounds can pass through the human digestive system without being metabolized and their concentration estimates can be as much as tens of mg/L in the human urine [18, 19] and even hundreds of mg/L in the human gastrointestinal tract [20, 21]. With metabolically inert structures, these nonnutritive sweeteners can be directly excreted to the environment from the human body. Currently these compounds are frequently detected in subsurface water, groundwater, and especially, wastewater treatment plants (WWTPs). The concentrations of sweeteners detected can be up to 2.5 mg/L in effluents from WWTPs [22, 23].

Although nonnutritive sweeteners have been developed to allow individuals to avoid the negative effects related to common sugars, a few commonly used nonnutritive sweeteners have recently been associated with health risks. For instance, in vitro tests demonstrated that nonnutritive sweeteners (saccharin (SAC), sucralose (SUC), and aspartame (ASP)) can induce the formation of urinary bladder tumors [24]. In addition, nonnutritive sweeteners are associated with the occurrence of glucose intolerance, which is thought to occur through alterations in the gut microbiota [25], and there is evidence that SAC, SUC, ASP, and acesulfame potassium (ACE-K) cause DNA damage in bacteria [26, 27]. The latter would likely activate the DNA damage response system (SOS response) of bacteria for remediating the damage. Conjugative ARG transfer has been indicated to be related to the SOS response [28]. Therefore, we hypothesize that these nonnutritive sweeteners may enhance the horizontal transfer of ARGs.

In this study, we tested our hypothesis that the commonly used nonnutritive sweeteners play a role in the spread of AMR. We selected four widely used nonnutritive sweeteners (SAC, SUC, ASP, and ACE-K) to test this hypothesis. We established three model conjugation systems to investigate whether these sweeteners promoted plasmid-mediated conjugative transfer in both environmental and clinical settings. In addition, the dynamic conjugation process at the single-cell level was visualized in real time through microfluidics and confocal microscopy. The underlying mechanisms of conjugative transfer were inferred from changes detected in reactive oxygen species (ROS) production, the SOS response, cell membrane permeability, and transcriptomic responses. These findings show that nonnutritive sweeteners pose an underappreciated ecological risk because they can promote the dissemination of ARGs.

## Methods

### Bacterial strains, growth conditions, and nonnutritive sweeteners

Donor strains *E. coli* K-12 LE392 and *E. coli* K-12 MG1655 harbor the plasmid RP4 (carries the ampicillin (Amp), kanamycin (Kan), and tetracycline (Tet) resistance genes) and the plasmid pMS6198A (carries the ampicillin (Amp) resistance gene), respectively. While recipient strains *E. coli* K-12 MG1655 and *P. alloputida*, both of which are resistant to chloramphenicol (Chl), were selected as recipients for the conjugation within and across genera, respectively. *E. coli* strain J53, which is resistent to sodium azide, was also used as another recipient. Strains were cultivated in Luria-Bertani (LB) broth that contained 100 mg/L Amp for the donors, 10 mg/L Chl for *P. alloputida*, 17 mg/L Chl for *E. coli* K-12 MG1655, and 100 mg/L sodium azide for *E. coli* J53. Four nonnutritive sweeteners, saccharine (SAC), sucralose (SUC), aspartame (ASP), and acesulfame potassium (ACE-K) used in this study, were purchased from Sigma-Aldrich (USA).

### Determination of MICs against antibiotics

Plate-reader measurements of bacterial growth in the presence of various antibiotic concentrations were used for MIC values. For the strains used in conjugative experiments (both within and across genera) as well as the post-conjugation transconjugants, all dose responses for the four antibiotics (Amp, Kan, Chl, and Tet) were quantified as previously described [29]. Initially, the strains were inoculated in LB media and exponential growth was obtained. Cell concentrations were adjusted to about 10^5^ CFU/mL using LB media and then transferred to 96-well plates. For each well containing 75 µL LB media with different levels of antibiotics, 75 µL of cell suspensions were added. The plates were then incubated at 30 °C for 20 h before measuring the optical density (OD) by a plate reader at a wavelength of 600 nm (Tecan Infinite M200, Swiss). Three replicates per concentration of all antibiotics were prepared and the control containing only sterilized Milli-Q water was simultaneously prepared. MIC_90_, determined as the antibiotic concentration, at which 90% of cell growth is inhibited, was used in this study.

### Mobile plasmid-mediated conjugative transfer models

Since our aim was to unravel whether and how the tested sweeteners (water soluble) would affect the conjugation dynamics in water environment, rather than soil-like environment, we used liquid media (rather than solid media) to do conjugation assays. In this study, three conjugative transfer models were established to evaluate the effects of nonnutritive sweeteners on the conjugative transfer of ARGs. Model I consisted of intra- and inter-genera conjugation process under environmental setting. For the intragenus conjugative transfer, the recipient was selected on the LB agar plates containing 17 mg/L Chl. The intragenus transconjugants were selected on the LB agar plates containing 100 mg/L Amp, 33 mg/L Kan, 17 mg/L Chl, and 20 mg/L Tet. For the intergenus conjugative transfer, the recipient (*P. alloputida*) was selected from LB broth media containing 10 mg/L Chl. The intergenus transconjugants were selected from LB agar plates containing 100 mg/L Amp, 33 mg/L Kan, 10 mg/L Chl, and 20 mg/L Tet.

After culturing in LB broth media overnight, the bacteria were centrifuged at 5000 rpm for 5 min and washed by substrate-free phosphate-buffered saline (PBS) two times in order to remove the culture media and antibiotic residues. The collected bacteria were then suspended in PBS. The mating system for the conjugative transfer was conducted in a PBS-based solution and consisted of both donor and recipient bacteria at cell densities of 10^8^ CFU/mL with a donor/recipient ratio of 1:1. The mixture (total volume of 1 mL) was then exposed to different concentrations (0, 0.003, 0.03, 0.3, 3, 30, and 300 mg/L) of the four tested nonnutritive sweeteners. These levels of sweeteners cover both environmental and clinical relevant range and were lower than MIC concentrations for the bacteria used in this study (Table S1). Following addition of sweeteners, the mating systems were well mixed by a vortex and subjected to 8-h incubation at 25 °C (the mating process). The system was then mixed again and then poured onto LB agar-selective plates containing antibiotics (prior to selection plating, 100 times dilutions were made for the intragenus conjugation, while no dilution was made for the intergenus conjugation). Separate preparations of the donor and recipients were simultaneously poured onto the transconjugant selective plates in order to measure the background mutation of strains resulting in antibiotic resistance. The plates were incubated at 30 °C for 48 h and then counting of transconjugants and recipients was performed separately. Moreover, the reverse conjugation experiment was also conducted (see the details in Text S1).

Model II was set up to test the conjugation process under clinically relevant condition by using clinical relevant plasmid pMS6198A (isolated from the urine of a patient suffering from a urinary tract infection, UTI [30]). The donor *E. coli* K-12 MG1655 containing pMS6198A plasmid with Amp resistance gene and recipient *E. coli* J53 containing resistance to sodium azide were used. Experiments were conducted following the similar procedures as Model I but the mating medium (LB), time condition (37 °C for 2 h), and selective plates (LB agar plates containing 100 mg/L of both Amp and sodium azide) for transconjugants were different.

Model III referred to the single-cell level analysis based on microfluidic system (Text S2). Briefly, overnight cultures of donor (*P. alloputida* containing *gfp*-RP4 plasmid; *dsRed/GFP-lacI*^*q*^, red fluorescence) and recipient (wild type of *P. alloputida*; no fluorescence but green only when the conjugative plasmid was received) were diluted tenfold and were incubated in LB broth media with shaking at 30 °C for 2 h. Afterward, cell suspensions were washed with PBS solution to remove any LB broth residue and were adjusted to an OD_600_ comprised between 0.7 and 0.8. In total, 300 µL of both donor and recipient cells were mixed (1:1 ratio, v/v) and vortexed before loading onto the B04A microfluidic plate chamber (ONIX, CellASIC®, Merck) with 5 psi for 1 min. Simultaneously, four nonnutritive sweetener solutions were loaded at 1 psi and the temperature was maintained at 25 °C. In total, 100 µM IPTG was also supplied. Cells were imaged at different time intervals for 2–3 h.

### Measurement of reactive oxygen species (ROS)

To understand whether oxidative stress has an effect on ARG’s transfer, intracellular ROS concentrations were measured using the 2’,7’-dichlorofluorescein diacetate (DCFDA) cellular ROS detection assay kit (Abcam, UK). Initially, the cell suspensions (initial concentration of about 10^6^ CFU/mL in PBS solution) were incubated with DCFDA at 20 μM for 30 min, at 37 °C in the dark. The sweeteners were then added and incubated for 2 h at room temperature in the dark. After that, the suspensions were analyzed by a CytoFLEX S flow cytometer (Beckman Colter, USA) with excitation at 488 nm and emission at 525 nm. Both positive (3% hydrogen peroxide, final concentration) and negative (Milli-Q water) suspensions were used as controls in the ROS analysis.

### Measurement of cell membrane permeability

The condition of cell membranes was examined to explore how they may contribute to the gene transfer. Cell membrane permeability of both donors and recipients was determined using the propidium iodide (PI, Life Technologies, USA) dye at the final concentration of 20 µM, according to the described method [12, 31]. Typically, bacterial cell suspensions of ≈10^6^ CFU/mL in PBS solution were obtained after washing the collected cells that were cultured in LB liquid overnight. Then, for each test, 100 μL of cell solution was added to the 2 mL tubes containing different levels of one of the four sweeteners. These tubes were put at room temperature (25 °C) for 2 h. A control of damaged cells was prepared as described above that was heated at 80 °C for 2 h. Another control of cells was prepared as above, except it had no sweetener treatment. Following the treatments, 1 μL of PI dye (2 mM) was added to the above cell solutions and incubated in the dark for 15 min at 25 °C. Samples of each cell preparation were applied to the flow cytometer that used 488 nm-excitation and 561 nm-emission detection. All samples were prepared in biological triplicates and technical duplicates.

### Plasmid extraction, gel electrophoresis, and PCR assays for resistance genes

The donors *E. coli* K-12 LE392 (RP4) and *E. coli* K-12 MG1655 (pMS6198A), the recipients *P. alloputida*, *E. coli* K-12 MG1655, and *E. coli* J53, as well as transconjugants (obtained from the transconjugant-selective plates) were cultured in LB broth overnight at 30 °C. Plasmids were extracted from the prepared cultures using the GeneJET Plasmid Miniprep Kit (Life Technologies, Australia), according to the manufacturer’s instructions. Agarose gel electrophoresis was used to confirm the presence of plasmids in the transconjugants. Further, qualitative PCR was employed to confirm whether the amplified ARGs in the transconjugants were the same to their corresponding plasmids (RP4 or pMSA6198). The PCR assays were carried out in a 20-μL volume reaction, which contained 1 μL of DNA sample, 10 μL of Platinum Green Hot Start PCR 2× Master Mix (Invitrogen by Thermo Fisher Scientific), 2 μL of Platinum GC Enhancer, 0.2 μL of forward and reverse primers, and 6.6 μL of Milli-Q water. The information about those primers was shown in Table S2. The amplification process was run on an Applied Biosystems machine in the following conditions: 94 °C for 10 min, followed by 30 cycles of 94 °C for 1 min, 60 °C for 1 min, and 72 °C for 1 min. This was followed by a final extension at 72 °C for 7 min. To confirm whether a specific ARG *bla*_*TEM-1*_ was successfully transferred from donor *E. coli* K-12 MG1655 to recipient *E. coli* J53, total DNA was extracted from the donor, recipient, and transconjugants (*E. coli* J53 with pMS6198A plasmid) using PowerWater^®^ DNA extraction kit (MoBio), according to the manufacturer’s instruction. Afterward, PCR assays were conducted as described above. Visualization of all bands was conducted by SYBR safe DNA gel staining and the GeneRuler 1 kb DNA ladder.

### RNA extraction, genome-wide RNA sequencing, and transcriptomic analysis

The intergenera conjugation between *E. coli* K-12 LE392 and *P. alloputida* was set up as described above. The mating systems were treated by four types of sweeteners at 0 and 3 mg/L for 2 h and were used for the RNA extractions. Each system was run in biological triplicates. The bacterial cell pellets were collected by centrifugation at 6000 rpm, 4 °C for 6 min. Total RNA was extracted from the pellets using the RNeasy Mini Kit (QIAGEN, Germany) based on the manufacturer’s instructions, with an additional cell lysis step of bead-beating. All RNA samples were then delivered to Macrogen Co. (Seoul, Korea) for construction of strand-specific cDNA library and Illumina sequencing of paired-end genome (HiSeq 2500, Illumina, USA) after passing their quality control. CummeRbund package in R was used to conduct the statistical analysis and visualization. The fragments per kilobase of a gene per million mapped reads (FPKM) was measured to quantify gene expression. The difference in gene expression between the control (without addition of any sweeteners) and the sweetener-treated groups was presented as log_2_ fold change (LFC). The significance of transcriptome data for expression of genes was determined by a false discovery rate (FDR)-adjusted *p* value less than 0.05. All samples were prepared in triplicates.

### Statistical analysis

All the experiments were conducted independently at least in biological triplicate. All phenotypic data were expressed as mean ± SD and were analyzed with SPSS 25.0 (SPSS, Chicago, USA). The results were analyzed by Analysis of variance (ANOVA) and Independent-sample *t* test methods, with the Benjamini–Hochberg correction. The corrected *p* values less than 0.05 were considered to be statistically significant.

## Results

### Nonnutritive sweeteners promote conjugative transfer

**Fig. 1:**
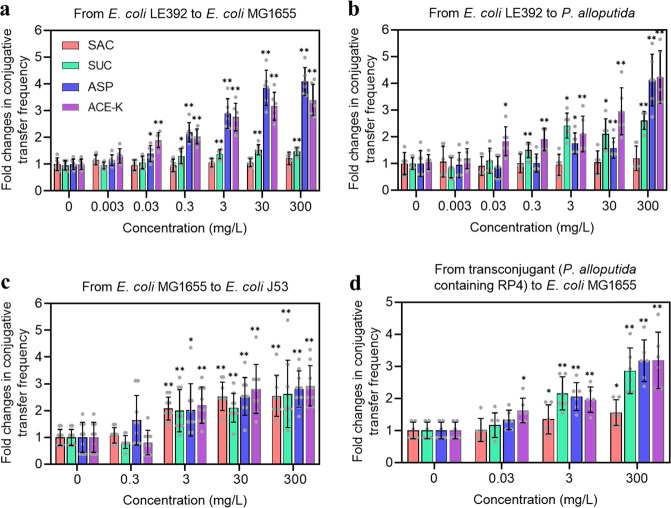
Nonnutritive sweeteners (SAC, SUC, ASP, and ACE-K) promoted RP4 plasmid-mediated conjugative transfer. **a** Fold changes in conjugative ARG transfer within genera. At high concentrations (>0.3 mg/L), all tested sweeteners (except for SAC) promoted conjugation (*N* = 6; ANOVA, *p* < 0.05). **b** Fold changes in conjugative ARG transfer across genera. All tested sweeteners except for SAC had positive effects on intergenus conjugative transfer (*N* = 6; ANOVA, *p* < 0.05). **c** Fold changes in conjugative ARG transfer from *E. coli* K-12 MG1655 to *E. coli* J53. All tested sweeteners at 3 mg/L or above significantly promoted conjugation (*N* = 9; ANOVA, *p* < 0.05). **d** Fold changes in conjugative ARG transfer (reverse) from transconjugant *P. alloputida* to *E. coli* K-12 MG1655. The presence of all tested sweeteners enhanced the reverse conjugation (*N* = 6). Significant differences between individual sweetener-treated groups and the control (0 mg/L sweeteners) were tested with the independent-sample *t* test: *p < 0.05 and **p < 0.01.

To test the effect of nonnutritive sweeteners on the conjugative transfer of ARGs, both intra- and intergenus-transfer experiments (model I) were first conducted, in which the bacteria were exposed to various concentrations of four commonly used nonnutritive sweeteners (SAC, SUC, ASP, and ACE-K) for 8 h at room temperature. Notably, in both mating systems, the whole concentration range (from 0.03 to 300 mg/L) of three sweeteners (SUC, ASP, and ACE-K) caused a significant concentration-dependent increase (*p* = 0.00017 ~ 0.047, Fig. S1a, b); Pearson correlation analysis was shown in Table S3 in conjugative transfer compared to the control (Fig. 1a, b). The intragenus (donor *Escherichia coli* K-12 LE392 and recipient *E. coli* K-12 MG1655) spontaneous conjugative transfer frequency was (1.9 ± 0.2) × 10^−4^ transconjugants per recipient cell (Fig. S2). However, the conjugative transfer frequencies were significantly enhanced by the sweeteners SUC, ASP, and ACE-K at 0.3 mg/L or above. For example, SUC, ASP, and ACE-K at 300 mg/L enhanced the conjugative frequencies by 1.5- (*p* = 0.00027), 4.1- (*p* = 0.000000089), and 3.4-fold (*p* = 0.0000020), respectively (Fig. 1a). In contrast, SAC did not significantly change the conjugative transfer frequency in the conjugation system (*p* = 0.200 ~ 0.670, Fig. 1a). For intergenus conjugation (donor *E. coli* K-12 LE392 and recipient *Pseudomonas alloputida*), all sweeteners at concentrations of 3 mg/L or higher (except for SAC) were seen to promote the conjugative transfer of the donor RP4 plasmid to recipients of different genera (*p* = 0.000047 ~0.042, Fig. 1b). SUC, ASP, and ACE-K at 300 mg/L caused a great increase in conjugative transfer, by 2.6- (*p* = 0.0000020), 4.1- (*p* = 0.000036), and 4.2-fold (*p* = 0.000019), respectively (Fig. 1b). It should be noted that the enhanced transfer frequencies were associated with the increased number of colonies on selective transconjugant plates, rather than decreased recipient numbers (Fig. S3).

To test whether conjugative transfer would be promoted by the tested compounds under clinical conditions, we used an IncA/C plasmid pMS6198A, which contains multiple clinically relevant resistance genes, in a uropathogenic *E. coli* strain isolated from a patient who suffered from a urinary tract infection [30] to conduct a conjugation experiment (model II, donor *E. coli* K-12 MG1655 and recipient *E. coli* J53) in liquid culture at 37 °C. Similarly, a significant concentration-dependent increase (*p* = 0.00017 ~ 0.047, Fig. S4 and Table S3) in pMS6198A transfer was also observed upon exposure to all four sweeteners (Fig. 1c and Fig. S5). This is different from the result in which SAC failed to enhance both intra- and intergenus conjugative transfer of the RP4 plasmid under environmental conditions.

The successful transfer of donor plasmids (RP4 and pMS6198A) to recipients was confirmed by a series of analyses. The MIC levels for transconjugants (recipient with transferred plasmid) were comparable or even higher than those for their corresponding parents that were resistant to all antibiotics (Fig. S6). In addition, plasmid extraction and PCR amplification of related resistance genes showed that all the transconjugants contained the same type of plasmid as the donor did and harbored the corresponding ARGs (Fig. S7). Thus, profiling of all the transconjugants confirmed that the donor plasmid was transferred to the recipient cells and conferred antibiotic resistance.

To understand whether transconjugants were capable of transferring ARGs to other candidates, we performed a reverse conjugation experiment (model III, donor *P. alloputida* containing the RP4 plasmid and recipient *E. coli* K-12 MG1655; Methods and Text S1) for 8 h at room temperature. Interestingly, this reverse conjugative transfer occurred and it was enhanced by all nonnutritive sweeteners (*p* = 0.000033 ~ 0.036, Fig. 1d; Fig. S8 and S9). Specifically, similar to the result of pMS6198A transfer, SAC significantly increased RP4 plasmid transfer (*p* = 0.036). Thus, the transferred RP4 plasmid in transconjugants maintained its mobility and could be transferred to other potential recipients. Collectively, these results demonstrate that the tested four sweeteners promoted plasmid-mediated conjugation in both nonpathogenic and pathogenic strains.

### Real-time visualization of conjugative plasmid transfer

**Fig. 2:**
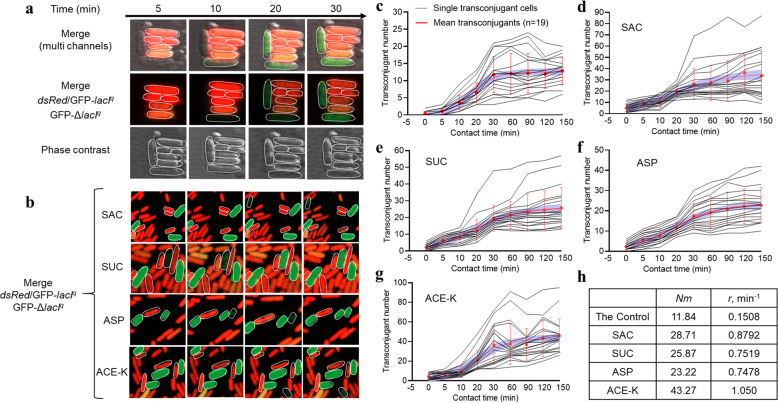
Real-time visualization of GFP-labeled RP4 plasmid transfer via conjugation at the single-cell level. **a**, **b** Time-lapse microscopy images of conjugation performed in a microfluidic chamber in the absence (**a**) or presence (**b**) of 300 mg/L of nonnutritive sweeteners. All scale bars indicate 1 µm. **c**–**g** Single-cell time-lapse quantification of transconjugant number in the control group as well as in the sweetener-treated groups (SAC, SUC, ASP, and ACE-K, respectively). Each black line represents the number of transconjugants produced at different time points. The red line is the fitting curve of the average transconjugant number with standard deviations (*n* cells analyzed). The violet area shows the 95% confidence interval. **h** Model fitting results of (**c**–**g**). N_m_, predicted maximum number of transconjugants; r, conjugation rate (transconjugants per min).

The spread of ARGs by conjugation is a dynamic process. However, clear real-time visualization of the cellular dynamics of conjugative transfer has yet to be obtained. Specifically, little is known about whether nonnutritive sweeteners could speed up the transfer of conjugative plasmids at the single-cell level. To achieve this, we developed an experimental system that is capable of real-time visualization of RP4 plasmid transfer by conjugation at the single-cell level using a fluorescence labeling technique (Methods and Text S2). This method could be useful or inspirational to other ecological studies, such as evolution of antibiotic resistance at a community-wide level. Time-lapse microscopy images showed that in the control group (without the addition of any sweeteners), the RP4 plasmid was successfully transferred to the recipient after 5 min of contact (Fig. 2a; Movie S1). In contrast, under exposure to four sweeteners, the plasmid acquisition process was accelerated, and successful transfer was observed within 5 min (Fig. 2b; Movie S2). Moreover, the number of transconjugants within 150 min were significantly increased under exposure to the tested sweeteners compared to the control (Fig. 2c–g). This was also mathematically supported by single-cell quantitative analysis, which showed higher transfer rates (r) under exposure to the tested sweeteners than in the control (Fig. 2h). A 6.0-, 5.0-, 5.0-, and 7.0-fold increase of transfer rate was induced by SAC, SUC, ASP, and ACE-K, respectively. The fitted maximum number of transconjugants (N_m_) showed a more than 2.0- to 4.0-fold increase under treatment with these sweeteners. These results further confirm that nonnutritive sweeteners promote plasmid-mediated conjugation at the single-cell level.

### Nonnutritive sweeteners enhance ROS production and the SOS response

**Fig. 3:**
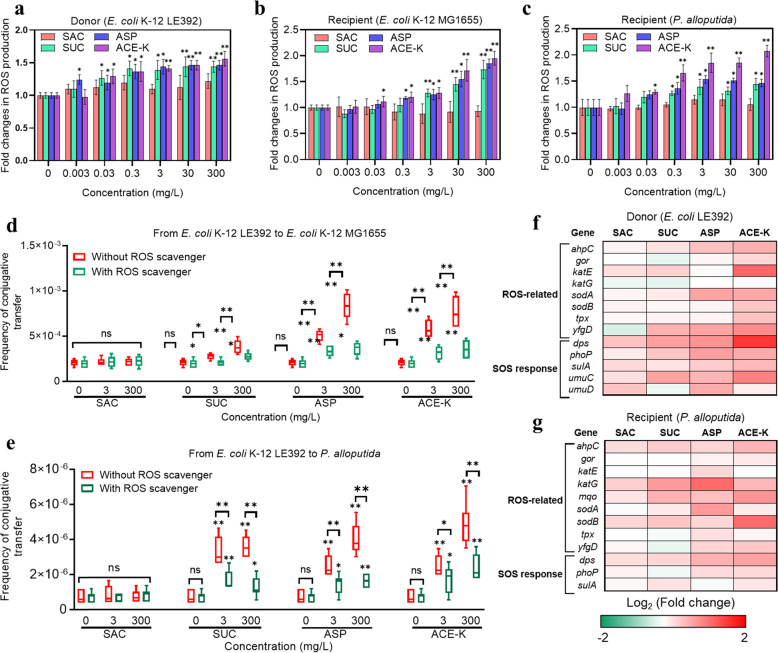
Nonnutritive sweeteners induced significant changes in ROS production. **a** Fold changes in ROS production in the donor *E. coli* K-12 LE392. **b** Fold changes in ROS production in the recipient *E. coli* K-12 MG1655 (*N* = 3). **c** Fold changes in ROS production in the recipient *P. alloputida* (*N* = 3). **d** Frequency of conjugative transfer within genera in the presence and absence of the ROS scavenger (*N* = 6). **e** Frequency of conjugative transfer across genera in the presence or absence of the ROS scavenger (*N* = 6). **f** Fold changes in the expression level of ROS and SOS response-related mRNA genes in the donor strain (*N* = 3). **g** Fold changes in the expression level of ROS and SOS response-related mRNA genes in the recipient strain (*P. alloputida*) (*N* = 3). Significant differences (**a**–**e**) between sweetener-treated groups and the control group were tested with an independent-sample *t* test and are indicated by *p < 0.05 and **p < 0.01. All genes shown in the figure were significantly (*p*_*adj*_ < 0.05) up- or downregulated under exposure to at least one of the tested compounds.

It has been reported that nonnutritive sweeteners can cause DNA damage [26] and induce oxidative stress in bacteria [32]. Thus, we measured ROS production in cells exposed to the tested sweeteners. Notably, in the strains treated with the sweeteners (except for SAC), concentration-dependent increase in ROS was detected (Fig. 3a–c; Fig. S10). For example, under treatment with the sweeteners (except SAC) at 300 mg/L, the relative fold change in ROS production in the donor *E. coli* K-12 LE392 increased 1.5-fold (*p* = 0.0010 ~ 0.0070, Fig. 3a). This increase was also observed when both the recipients *E. coli* K-12 MG1655 and *P. alloputida* were treated with these sweeteners (Fig. 3b, c). In contrast, exposure to the sweetener SAC did not result in any increase in ROS production in these donor and recipient strains. To further verify the promotion of ROS production by these compounds, thiourea, a scavenger of oxygen-free radicals [33, 34], was added to the mating system together with nonnutritive sweeteners. As expected, significant decrease in ROS production was detected in the three strains treated with SUC, ASP, and ACE-K after thiourea was added (Fig. S11).

To determine whether the increased production of ROS induced by these sweeteners promotes conjugative transfer, thiourea was added to both the intra- and intergenus conjugative systems, together with the sweeteners. Compared to the group without thiourea in both conjugative systems, addition of the scavenger caused no change in the conjugative transfer frequency in the SAC-treated group but significantly decreased the transfer frequency in the SUC-, ASP-, and ACE-K-treated groups (*p* = 0.000033 ~0.035, Fig. 3d, e). These results suggest that ROS production plays a significant role in the plasmid-mediated conjugation process.

Under oxidative stress caused by the tested sweeteners, bacterial defense may include changed expression of ROS-related genes (or ROS detoxification) as well as changed expression of SOS response-related genes. To track the expression of genes related to ROS production and the SOS response regulated by the tested sweeteners, the mRNA transcription level of genes from both the donor *E. coli* K-12 LE392 and recipient *P. alloputida* was measured. The transcripts for genes encoding enzymes involved in ROS detoxification in the donor were increased under exposure to the tested sweeteners (Fig. 3f; Table S4). For example, the gene encoding superoxide dismutase (*sodA*) exhibited a 1.3- (*p* = 0.032), 1.8- (*p* = 0.0052), and 1.8-fold (*p* = 0.0023) increase in expression in the donor treated with SUC, ASP, and ACE-K, respectively. Accordingly, an SOS response was detected in the donor after exposure to these sweeteners (Fig. 3f; Table S5). Increased transcription was detected for the genes *sulA* (1.3- to 1.6-fold change, *p* = 0.0040 ~ 0.036) and *umuC* (1.6- to 2.1-fold change, *p* = 0.014 ~ 0.040) after treatment with the three sweeteners. We also found increased expression of ROS detoxification- and SOS response-related genes in recipients treated with SUC, ASP, and ACE-K (Fig. 3g; Tables S6 and S7). In contrast, SAC treatment did not result in significant regulation of the genes mentioned above in the donor or recipient.

It is apparent that SUC, ASP, and ACE-K increased ROS production in both intra- and intergenus conjugative systems, whereas SAC did not induce any change in ROS production in the strains tested. We propose that nonnutritive sweeteners induced ROS overproduction and increased ROS detoxification, and correspondingly stimulated oxidative stress responses (the SOS response) in the strains, eventually contributing to the promotion of intra- and intergenus conjugative transfer.

### Nonnutritive sweeteners increased cell membrane permeability

It is worth noting that addition of the scavenger thiourea did not completely reverse the increase in the frequency of conjugative transfer caused by sweeteners (i.e., SUC, ASP, and ACE-K). For example, after addition of thiourea to the mating system that was exposed to 300 mg/L ACE-K, the frequency of conjugative intragenus transfer was (3.5 ± 0.4) × 10^–4^ transconjugants per recipient cell, which was still significantly (*p* = 0.0090) higher than the value for the control ((2.0 ± 0.3) × 10^–4^ transconjugants per recipient cell, Fig. 3d). This indicated that ROS production is not the only mechanism for conjugation.

**Fig. 4:**
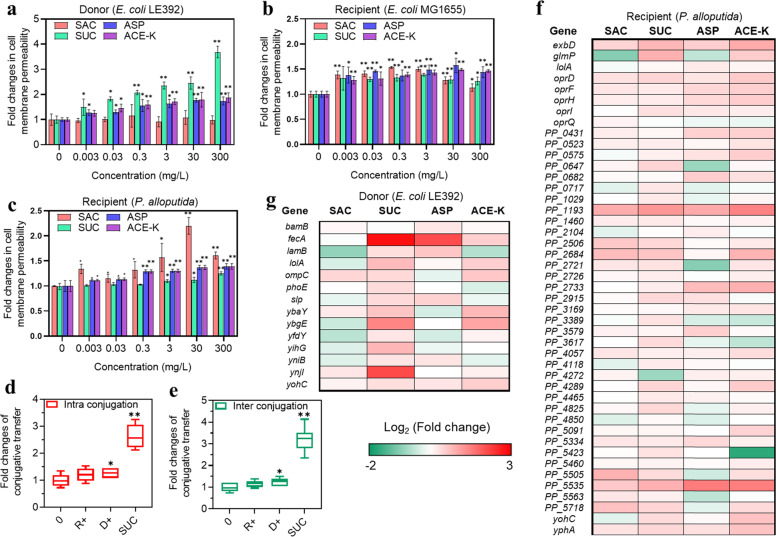
Changes related to cell membrane permeability detected in the bacterial conjugation system after exposure to nonnutritive sweeteners. **a** Fold changes in cell membrane permeability in the donor *E. coli* K-12 LE392 (*N* = 3). **b** Fold changes in cell membrane permeability in recipient *E. coli* K-12 MG1655 (*N* = 3). **c** Fold changes in cell membrane permeability in recipient *P. alloputida* (*N* = 3). **d**, **e** Influence of pre-exposure of the donor (D) and two recipients (R) to SUC on the fold changes in intra- and intergenus conjugative transfer (*N* = 6 and 9, respectively). R+ indicates that the recipient was pre-exposed to 3 mg/L SUC for 2 h before conjugation experiment; D+ indicates that the donor was pre-exposed to 3 mg/L SUC for 2 h; SUC indicates that the mixture of the donor and the recipient was directly exposed to 3 mg/L SUC for 8 h of conjugation without pre-exposure. **f** Fold changes in relative expression levels (mRNA) of cell membrane-related genes in the donor strain (*N* = 3). **g** Fold changes in relative expression levels of cell membrane-related genes in the recipient strain (*P. alloputida*) (*N* = 3). Significant differences between sweetener-treated groups and the control group were tested with an independent-sample *t* test and are indicated by * for *p* < 0.05 and ** for *p* < 0.01. All genes shown in the figure were significantly (*p*_*adj*_ < 0.05) up- or downregulated under exposure to at least one of the tested compounds.

Bacterial cell membranes act as a barrier against horizontal transfer of ARGs [11], and the permeability of this barrier could play a significant role in the transport of substances into or outside of cells. Therefore, we tested whether nonnutritive sweeteners could change the cell membrane permeability. To examine this concept, we treated both the donor *E. coli* LE392 and the two recipients (*E. coli* K-12 MG1655 and *P. alloputida*) with different concentrations of the four sweeteners. After 2 h of treatment, the cell membrane permeability was increased by up to 3.7-fold (*p* = 0.000047 ~ 0.0019) in the donor by all the sweeteners (300 mg/L) except SAC (Fig. 4a; Fig. S12). This increase was also observed in the two recipients treated with all sweeteners (Fig. 4b, c), exhibiting an increase of up to 1.5- and 1.6-fold, respectively (*p* = 0.0010 ~ 0.028), under exposure to all tested sweeteners at 300 mg/L. In addition, we found that SAC, together with the other three sweeteners, significantly increased the permeability of another donor strain, *E. coli* K-12 MG1655 (*p* = 0.00024 ~ 0.0082, Fig. S13a). Similar results were also found for the permeability of the recipient strain *E. coli* J53 (*p* = 0.000088 ~ 0.0074, Fig. S13b).

Our results showed that the SAC failed to enhance ARG transfer from *E. coli* LE392 to either *E. coli* K-12 MG1655 and *P. alloputida* but enhanced conjugative transfer between *E. coli* K-12 MG1655 and *E. coli* J53, as well as between the strain *P. alloputida* containing the RP4 plasmid and the strain *E. coli* K-12 MG1655 (i.e., reverse conjugation). This raises the question of whether the permeability of the donor or recipient (or both) is important for conjugative transfer. To this end, we performed different pre-exposure to separately increase the cell membrane permeability of the donor (*E. coli* LE392) and two recipients (*E. coli* K-12 MG1655 and *P. alloputida*) before mixing for conjugation experiments (Text S3; Table S8). We treated each strain with 3 mg/L SUC, which significantly increased the permeability of the donor and recipient (Fig. 4a–c). The results showed that there was no significant change in conjugation frequency when the recipients were pre-exposed to SUC (*p* = 0.078 ~ 0.19, Fig. 4d, e; Fig. S14). This indicates that the increased permeability of the recipient did not guarantee enhanced conjugative transfer. In contrast, both intra- and intergenus conjugation frequencies were significantly (*p* = 0.00000000034 ~ 0.040) promoted when the permeability of the donor was significantly increased. Overall, these results indicate that cell membrane permeability is positively mediated by nonnutritive sweeteners in mating systems and that donor permeability plays a more critical role than recipient permeability in the conjugation process.

We tested whether genes that mediate membrane permeability at the molecular level were upregulated in the donor and recipient (*P. alloputida*) after treatment with the tested sweeteners at 3 mg/L. Genes related to cell membrane permeability in both donor and recipient cells were upregulated after exposure to the tested sweeteners (Fig. 4f, g). For instance, *fecA*, which encodes an outer membrane transporter, and *ybgE*, which encodes an inner membrane protein, were significantly upregulated (1.5- to 7.0-fold (*p* = 0.0020 ~0.037) and up to 2.7-fold (*p* = 0.0060 ~0.044), respectively, Table S9) in the donor cells treated with SUC, ASP, and ACE-K (Fig. 4f). In contrast, SAC did not induce significant changes in the expression of cell membrane-related genes in the donor. These findings are consistent with the results showing that all sweeteners, except SAC, increased the membrane permeability of the donor. Nevertheless, cell membrane-related genes in the SAC-treated recipient showed increased expression, as seen in the other sweetener-treated recipient groups (Fig. 4g; Table S10).

These results confirm that the tested sweeteners increased the expression of cell membrane-related genes, and then increased the cell membrane permeability of the donors or both parents to promote the conjugative transfer of plasmids between strains.

### Nonnutritive sweeteners upregulate the expression of conjugation-related genes on the RP4 plasmid

**Fig. 5:**
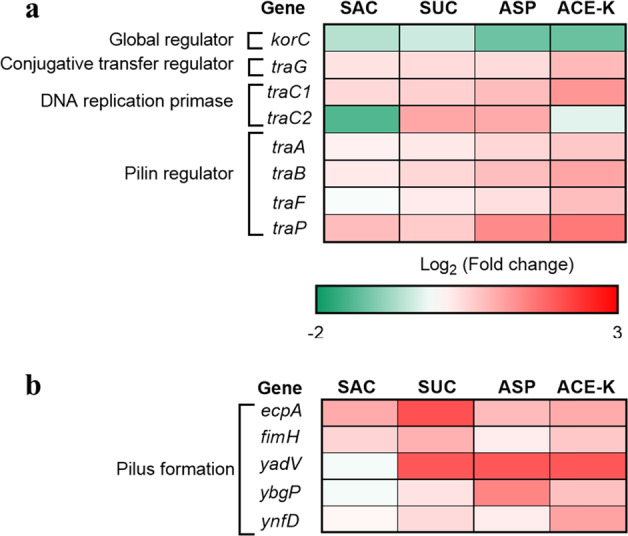
Transcriptional analysis of conjugation-related core genes expression. **a** Fold changes in expression of genes in conjugative RP4 plasmid. **b** Fold changes in expression of genes responsible for pilin channel in donor cell. A 3 mg/L of each nonnutritive sweetener was used for the treatment. All genes shown in the figures were significantly (*p*_*adj*_ < 0.05) up- or down-regulated under exposure to at least one of the tested compounds.

The process of conjugative transfer mediated by the RP4 plasmid requires the regulation of global regulatory genes and conjugative transfer-related genes [35, 36]. After treatment with 3 mg/L SUC, ASP, and ACE-K, we found that the core global regulatory gene *korC* was repressed in comparison with the control group. Accordingly, the conjugative transfer regulator gene *traG* (which connects the relaxosome and mating pair formation complex) and DNA transfer and replication (Dtr) genes *traC1* and *traC2* exhibited increased expression compared with the mRNA expression detected in the control group (Fig. 5). During the conjugation process, the plasmid is thought to be transferred via a pilin bridge between donor and recipient cells [35]. The expression levels of pilin formation-related genes, including *traA*, *traB*, *traF,* and *trap*, were also upregulated in the sweetener-treated groups (Fig. 5). These genes also contribute to the formation of the mating pair formation (Mpf) system. For example, compared to the control, mRNA expression of gene *traB* (encoding a conjugative transfer protein) was significantly upregulated by more than 1.5- (*p* = 0.0046), 1.4- (*p* = 0.047), and 2.1-fold (*p* = 0.000050) under exposure to SUC, ASP, and ACE-K, respectively (Fig. 5a; Table S11). Together, these results reveal that these sweeteners (except for SAC) cause increased mRNA expression levels of genes related to RP4 plasmid transfer and replication (Dtr genes) and then promote the formation of the pilus channel in the Mpf system to allow RP4 plasmid transfer from the donor to the recipient.

The conjugation process also requires direct cell-to-cell contact. This can be achieved by the periplasmic pilin of donor cells. In the donor *E. coli* K-12 strain, pili operons have been found to play roles in cell adhesion and colonization [37]. In the present study, the expression of genes in these adhesion-relevant operons was upregulated. For example, the expression of gene *ecpA* was upregulated by 1.8- (*p* = 0.035), 4.2- (*p* = 0.0070), and 2.1-fold (*p* = 0.033) after treatment with SUC, ASP, and ACE-K, respectively (Fig. 5b; Table S12).

## Discussion

Based on the three established conjugation models and a real-time dynamic conjugation system at the single-cell level, our work for the first time demonstrates that four commonly used nonnutritive sweeteners can promote ARG transfer by conjugation at environmentally and clinically relevant concentrations. Multiple pieces of evidence were gathered, including from measurement of ROS production, cell membrane permeability, and whole-genome RNA sequencing analysis, to elucidate the underlying mechanisms. These measurements, coupled with the real-time dynamic analysis of conjugation process, enable the identification of key factors in HGT and quantitative estimates of the extent of HGT in diverse microbial communities. Although the enhanced conjugative frequency could be significantly decreased after adding the scavenger thiourea, nonnutritive sweetener (SUC, ASP, and ACE-K)-treated groups still showed higher frequencies of conjugative transfer than the control group. These results suggest that ROS production induced by nonnutritive sweeteners plays a significant role in the promotion of conjugative transfer but is not the only underlying mechanism.

As a permeable barrier controlling the entry of extracellular substances, the cell membrane also plays a role in the conjugative transfer of ARGs [12, 38, 39]. Our results demonstrate that in addition to increased ROS production, cell membrane permeability, especially that of the donor, also plays an important role in the conjugative transfer frequency of the RP4 plasmid. Specifically, SAC increased the cell membrane permeability of both recipients (*E. coli* MG1655 and *P. alloputida*) but failed to increase that of the donor *E. coli* LE392. This might be why SAC did not increase the conjugative transfer frequency of the RP4 plasmid in mating model I. In contrast, SAC can increase the conjugative transfer frequency of the RP4 plasmid (reverse conjugation), the GFP-labeled RP4 plasmid in *P. alloputida*, and the pMS6198A plasmid, since the cell membrane permeability of the donor *P. alloputida* (Fig. 4c) or *E. coli* K-12 MG1655 (Fig. S13a) increased significantly. We further verified this hypothesis, based on experiments, with the exposure of different donors and recipients to nonnutritive sweeteners. When the permeability of the donor was increased but that of the recipient was not, conjugative transfer was significantly promoted. In contrast, when the permeability of the recipients was increased but that of the donor was not, both intra- and intergenus conjugative transfer showed insignificant changes. It has been reported that in the transfer of ARGs, donors with high expression of the conjugation machinery were shown to be associated with low-receptivity recipients [40]. Thus, the increased permeability of the donor may cause increased ARG transfer to the recipient and result in increased conjugative transfer frequency.

**Fig. 6:**
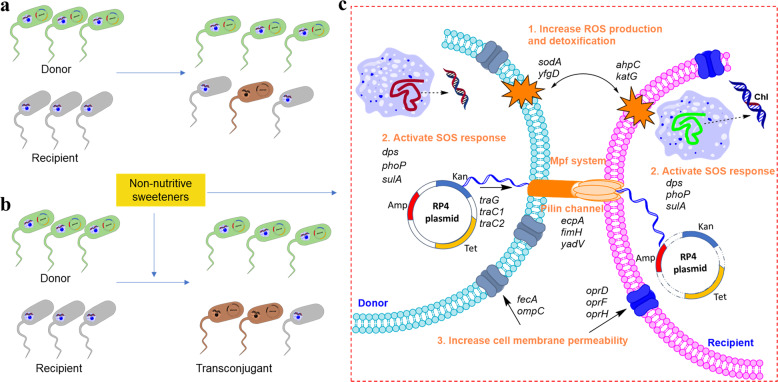
A model depicting the mechanisms underlying the RP4 plasmid-mediated conjugative transfer promoted by nonnutritive sweeteners. **a** Spontaneous conjugative transfer. **b** Enhancement of RP4 plasmid conjugative transfer by nonnutritive sweeteners. **c** Mechanism underlying the sweetener-induced conjugative transfer process: nonnutritive sweeteners significantly increase ROS production and detoxification, and cell membrane permeability in both donor and recipient strains. This increase triggers cell-to-cell contact between the donor and recipient through the mating pair formation system and a pilus channel. Then, these sweeteners activate conjugative RP4 plasmid replication and transfer through the pilin channel from the donor to the recipient.

As aforementioned, we suggest that the mechanisms (Fig. 6) underlying the tested sweetener-promoted conjugative transfer include increased ROS production and detoxification, and increased cell membrane permeability of the parent bacteria. In addition, the tested sweeteners promote increased expression levels of conjugative transfer-related genes and activate plasmid DNA processing (Tra1) and mating pair formation (Mpf or Tra2) to enhance the intercellular transfer of the RP4 plasmid through a pilus channel.

As the environment and human gut system are reservoirs of various microbes, the emergence of nonnutritive sweeteners can be a driving force to shape these microbial communities. Specifically, nonnutritive sweeteners might play a significant role in the dissemination of ARG transfer in both environmental and clinical settings.

In the environmental setting, although various techniques, including adsorption, advanced oxidation processes, and biological treatment, are used to remove nonnutritive sweeteners, the removal efficiency of these sweeteners is generally low (less than 30%) [41–43]. We also found that nonnutritive sweeteners used were metabolically resistant to the tested bacterial strains (*p* = 0.062 ~ 0.700; see in Text S4; Fig. S15). Noticeably, the concentrations of nonnutritive sweeteners used in this study were environmentally relevant (e.g., 0.03 ~ 3 mg/L) [44]. Moreover, previous studies found that WWTPs served as hotspots harboring antibiotic- resistant bacteria (ARB) and ARGs due to HGT among indigenous bacterial species [45, 46]. Upon exposure to these compounds, it is reasonable to assume that the transfer frequency of ARGs would be promoted in WWTPs. In addition, our results show that the ARGs on the RP4 plasmid from transconjugants can also be transferred to other candidates (Fig. 1d) and the transfer rate can be facilitated by the four commonly used nonnutritive sweeteners (Fig. 2). It is possible that these sweeteners could cause a cascading spread of ARGs in the WWTPs, thus facilitating increased development of antibiotic resistance in downstream environmental bacteria. However, it should be noted that our work highlighted the results of conjugation between pure cultures. Further studies could be carried out to test whether these sweeteners could promote conjugation in mixed culture-based systems (e.g., activated sludge). In addition, our work highlighted the results of an acute exposure (~8 h) to the tested four sweeteners, rather than a chronic exposure. Considering that these compounds are being soaringly consumed and can exist in the environment for a long period, a chronic exposure to the tested sweeteners may induce an accumulative effect on the spread of antibiotic resistance, as antibiotics or heavy metals did [47–49]. Thus, it is meaningful and necessary to study the long-term effect of these sweeteners at lower concentrations (e.g., ng/L) on the spread of antibiotic resistance.

On the other hand, our work provided evidence that the four commonly used nonnutritive sweeteners (at 3 mg/L or above) significantly promoted conjugative transfer of plasmid pMS6198A that was isolated from the urine of a patient suffering from UTI (Fig. 1c). Considering that the concentrations of the tested sweeteners can be higher than 30 mg/L in urine [18], our result indicates that these nonnutritive sweeteners might enhance plasmid conjugation in the human urinary system. In addition, it is estimated that the human gut microbiota is composed of as many as 10^14^ bacteria [50], which are involved in multiple interactions relevant to host health [51] and serve as a transporter [52] and even a reservoir of ARGs [53]. In fact, the levels of consumed nonnutritive sweeteners in the human gastrointestinal tract are likely to be far higher than the ones used in the present study [20, 21]. The highest concentration (300 mg/L) of these compounds is also within the dosage range for average daily intake (i.e., 5 mg/kg/d sucralose) suggested for an individual with a body weight of 60 kg by the U.S. FDA and is also lower than the threshold concentrations (>1000 mg/L) regulated by the Codex General Standard for Food Additives (CODEX STAN 192-1995). Moreover, our study did in vitro assays of plasmid conjugation driven by the tested sweeteners in PBS or LB media, which were not totally clinically relevant to the human urinary and gut system conditions. In the future, it is worthwhile to validate whether these nonnutritive sweeteners could promote conjugative plasmid transfer in animal or human gut microbiomes, which will provide more concrete evidence.

Collectively, the findings of the present study provide evidence that four commonly used nonnutritive sweeteners can promote the horizontal transfer of ARGs via conjugation in both environmental and clinical settings. The establishment of a real-time dynamic analysis of conjugation process in this study will advance the ecological studies such as the spread of AMR in diverse conditions. Considering the substantial application of these sweeteners in food industry (over 117,000 metric tons globally consumed per year), our findings are a wake-up call to start evaluating the potential antibiotic-like roles induced by nonnutritive sweeteners. In vivo assays could be further applied to test whether nonnutritive sweeteners are able to promote conjugation in human urinary and gut systems in the long-term run.

## Supplementary information

Supplementary Information

Move S1

Movir S2

## Data Availability

All RNA sequencing data have been deposited to the Gene Expression Omnibus of National Center for Biotechnology Information (NCBI) and are accessible through the GEO series (GSE139245).
