# Supplementary material for: Nonnutritive sweeteners can promote the dissemination of antibiotic resistance through conjugative gene transfer
Source: ISME J. 2021 Feb 15;15(7):2117–30. doi: 10.1038/s41396-021-00909-x (PMC8245538; doi:10.1038/s41396-021-00909-x)
Supplement: Supplementary file 1 — Supplementary Information [file 41396_2021_909_MOESM1_ESM.docx]

**Supplementary Information**

**Non-nutritive sweeteners can promote the dissemination of antibiotic resistance through conjugative gene transfer**

**Zhigang Yu^1^, Yue Wang^1^, Ji Lu^1^, Philip L. Bond^1^, Jianhua Guo^1,^***

**Affiliation**

^1^ Advanced Water Management Centre, The University of Queensland, St. Lucia, Brisbane, QLD 4072, Australia

* Corresponding author.

Email: [j.guo@awmc.uq.edu.au](mailto:j.guo@awmc.uq.edu.au)

This PDF file includes:

Supplementary Text S1 to S4

Figs. S1 to S15

Tables S1 to S12

Movies S1 to S2

References

**Text S1**

**Reverse conjugation**

Transconjugant (*P. alloputida* containing RP4 plasmid) collected from the intergenus conjugation experiment was used as donor cell. *E. coli* K-12 MG1655 that is resistant to Chl was selected as the recipient. The mating conditions were the same as described in the method of RP4 plasmid-mediated conjugative transfer systems. After 8 h mating time, cell suspensions were spread onto Difco^TM^ m-Endo Agar (capable of distinguishing *E. coli* and *P. alloputida* strains) selective plates that contained antibiotics, and then incubated at 30℃ for 48 h. After that, the colonies of transconjugants and recipients were separately enumerated and the transfer frequencies were calculated as described in the main text.

**Text S2**

**Microfluidics**

*Strains and culture.* *P. alloputida* strain carrying a GFP-encoded IncP-1α plasmid RP4 that contains resistance to Kan was used as the donor. The strain was also chromosomally tagged with a red fluorescence gene (*dsRed*) and lactose operon (*lacI^q^*) that represses the *gfp* gene expression. In this case, the donor cells emitted only red fluorescence (*dsRed/GFP-lacI^q^*). Recipient (wild type of *P. alloputida* strain) was nonfluorescent but was green fluorescent after the RP4 plasmid was received (i.e., recipient cell became transconjugant, *GFP-ΔlacI^q^*). Strains were cultivated in LB broth with or without 100 mg/L Kan and 100 *µ*M IPTG for donor or recipient.

*Image acquisition*. Conventional wide-field fluorescence microscopy imaging was carried out on an inverted laser scanning confocal microscope (ZEISS LSM 710, AxioObserver), which is equipped with Plan-Apochromat 40×/1.4 Oil DIC M27 objective and 3-channel QUASA spectral PMT array, and uses ZEN2.3 software for image acquisition. Acquisition was set up with 0.39 *µ*s pixel time of both GFP and *dsRed* fluorescence using a Fluo LED Spectra X light source. GFP fluorescence was detected at 488 nm excitation wavelength and 518 nm emission wavelength, while *dsRed* fluorescence was detected at 561 nm excitation wavelength and 585 nm emission wavelength. Each image contained 1024 × 1024 pixels.

*Modelling for data fitting*. Conjugation process requires cell-to-cell contact between donor and recipient cells. It takes time (several minutes) to achieve mating pairs before occurrence of conjugation [1]. Thus, we put forward three proposals related to conjugation process in microfluidic chamber: (1) lag time of conjugation process; (2) transfer phase of conjugation. After lag time, conjugative transfer starts from donor to recipient. Transfer rate is initially high because of lots of successful mating pairs (direct donor-recipient contact). Gradually, this rate decreases as the mating pair declines; and (3) limited conjugation process. The number of transconjugant could reach its the maximum (*Nm*), instead of always keeping an increase. A model below was used for conjugation dynamic of mobile plasmid between donor and recipient cells.

$\frac{dN}{dt}=r\left( 1-\frac{N}{N_{m}} \right)N$

where *N* is the number of transconjugant at time t; *N_m_* is the maximum number of transconjugant; *t* means the contact time for conjugation; *r* refers to the transfer rate (transconjugant per min).

**Text S3**

**Pre-exposure of donor and recipients with different exposure routes to non-nutritive sweeteners**

To investigate whether different pre-exposure of bacterial by chemicals affects the RP4 plasmid-mediated conjugative transfer and to rule out whether the increase of cell membrane permeability of either the donor or the recipient plays a significant role in the RP4 plasmid conjugative transfer, three means of pre-exposure were used in this study: (1) only the donor (*E. coli* LE392) exposure to non-nutritive sweeteners before mating; (2) only the recipient exposure to non-nutritive sweeteners before mating; and (3) both the donor and the recipient exposure to non-nutritive sweeteners before mating. According to the result in this study, we found that SUC, ASP, and ACE-K significantly increased the cell membrane permeability of both the donor and two recipients (Fig. 4a-c). Herein, SUC was used as a representative of non-nutritive sweeteners in the pre-exposure experiments to significantly increase the permeability of each strain.

First, cultures of the donor and the recipients were overnight (about 14 h) incubated at 30℃ and were then incubated (1% incubation) in LB media for further growing. The donor cell was grown in the presence of 100 mg/L Amp, 33 mg/L Kan and 20 mg/L Tet, while the two recipients were grown in the LB media with 34 mg/L and 17 mg/L Chl, respectively. Afterwards, the cell pellets were collected by centrifuge at 5000 rpm for 5 min and were washed by PBS two times. The cells were vortexed to resuspend in the PBS solution and were then adjusted to reach an OD600 value of 0.46 (donor *E. coli* K-12 LE392), 0.31 (recipient *E. coli* K-12 MG1655) and 0.53 (recipient *P. alloputida*). Each strain was then treated by 3 mg/L SUC for 2 h. Detailed information about pre-exposure was shown in Table S8. After that, the cell suspensions were centrifuged and washed to remove any SUC residue, and finally were resuspended in PBS solution. The conjugation (within and across genera) experiments were conducted by mixing the donor and the recipient, with their cell density ratio of 1:1. Both negative (no pre-exposure and no addition of SUC) and positive (no pre-exposure but addition of SUC) control were simultaneously set up. After 8 h mating, the numbers of the recipient and the transconjugant were counted by spreading a certain volume of mating systems to the selective LB agar plates that contained different types of antibiotics.

**Text S4**

**LC-MS analysis of non-nutritive sweeteners**

**(1) Samples preparation.** Preparation of samples from conjugation system was similar to conjugation experiment. Three strains suspensions (*E. coli* K-12 LE392, *E. coli* MG1655 and *P. alloputida*) were separately prepared with PBS and their cell density was approximately 10^8^ CFU/mL. Afterwards, cells were exposed to non-nutritive sweeteners for 8 h before collecting supernatant by filtration (0.22 *µ*m membrane filters).

The procedure for the extraction and purification of non-nutritive sweeteners from the collected supernatant was followed elsewhere[2, 3]. Samples were extracted by passage through Oasis HLB solid phase extraction (SPE) cartridges (6 cm^3^, 200 mg sorbent; Waters, Milford, MA). Prior to use, the cartridges were conditioned with 2×5 mL methanol and 2×5 mL of milli-Q water, and the samples were loaded at around 1 mL/min. This was followed by 30 min vacuum drying and then elution with 6 mL methanol. After that, 3 mL mixture of acetone, methanol, and ethyl acetate (2:2:1, v/v/v) and 3 mL of methanol containing 5% ammonia were used to elute the cartridges. The eluent was combined and concentrated to about 500 *µ*L under a gentle stream of nitrogen at 35℃, using a TurboVap® LV Evaporator (Caliper Life Sciences, Runcorn, UK).

**(2) Instrumental analysis.** Four non-nutritive sweeteners were analysed by an AB 4000 Qtrap (AB Sciex, Carlsbad, CA, USA), interfaced with an Agilent 1200 Series HPLC system (Santa Clara, CA, USA). The analytes were separated by a 4.6 × 150 mm, 5 mm particle Phenomenex Luna Phenyl-Hexyl column (Torrance, CA, USA). Methanol and milli-Q water containing 0.1% formic acid were used as mobile phases. Flow rate of the mobile phase was set at 1.0 mL/min and the injection volume was 20 *µ*L. The regression coefficients (r^2^) for 9-point calibration standards calculated by weighting quadratic regression were > 0.99 for all target analytes. A method blank was analysed before, during and after every batch of samples. The measured concentrations of four target analytes in the blanks were below the limit of quantitation (LOQ). The continuing calibration verification standards injected after every batch (n =15) of samples analysed showed recoveries at 101 ± 19%.

One sample was selected randomly for matrix spike and matrix spike duplicate (MSD) with each batch of samples. Target analytes and their corresponding internal standards were spiked at 10-50 ng and passed through the entire analytical procedure. The background concentrations of analytes were subtracted for the recoveries. The overall average recoveries (n = 3) of non-nutritive sweeteners in spiked matrices were 105 ± 15% for SPM.


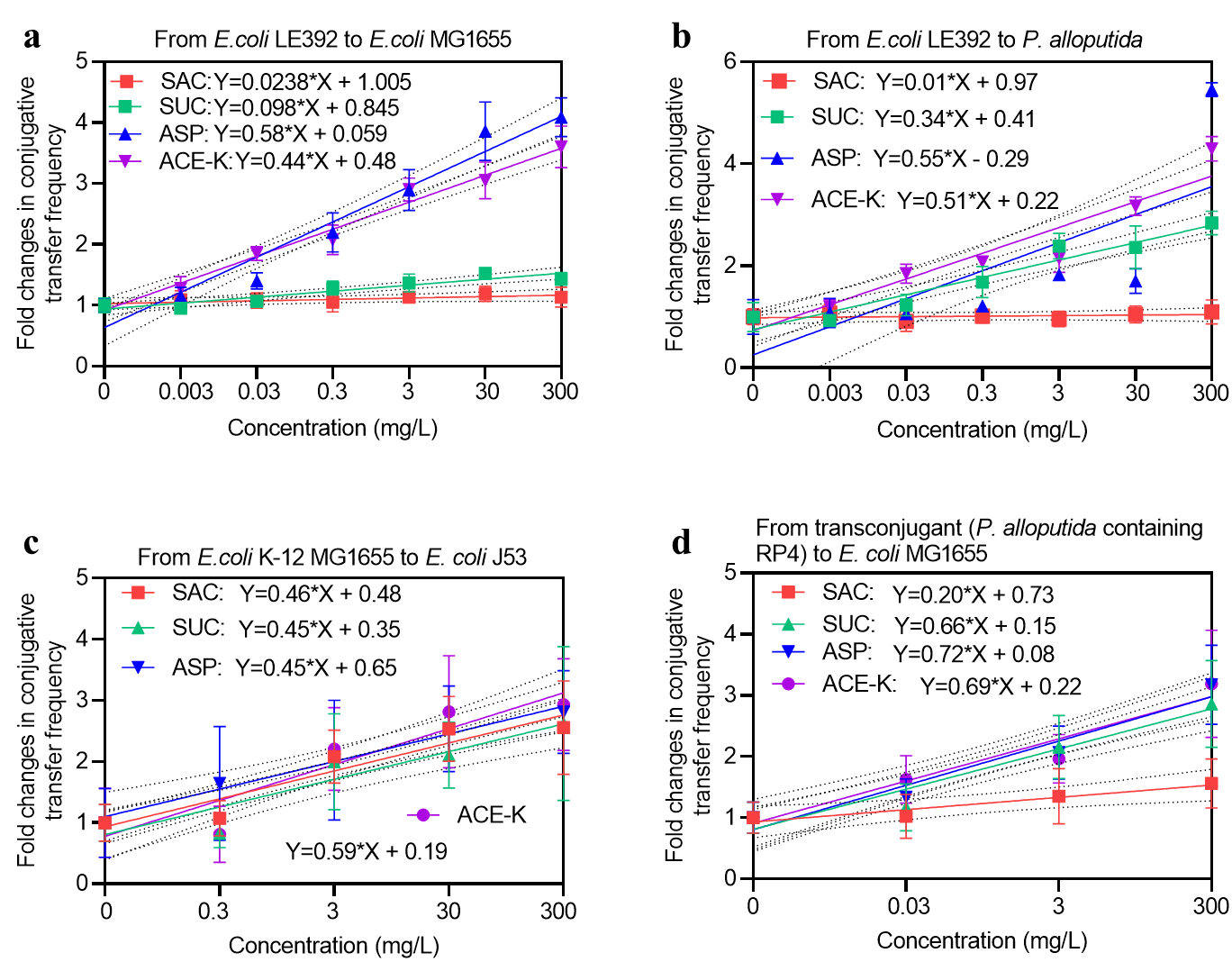


**Fig. S1.** Linear regression modelling of fold changes in conjugative transfer frequency (**a**, within; **b**, across). Results showed that there was a concentration-dependent increase pattern of fold changes, based on the significant non-zero of slope from each plot.


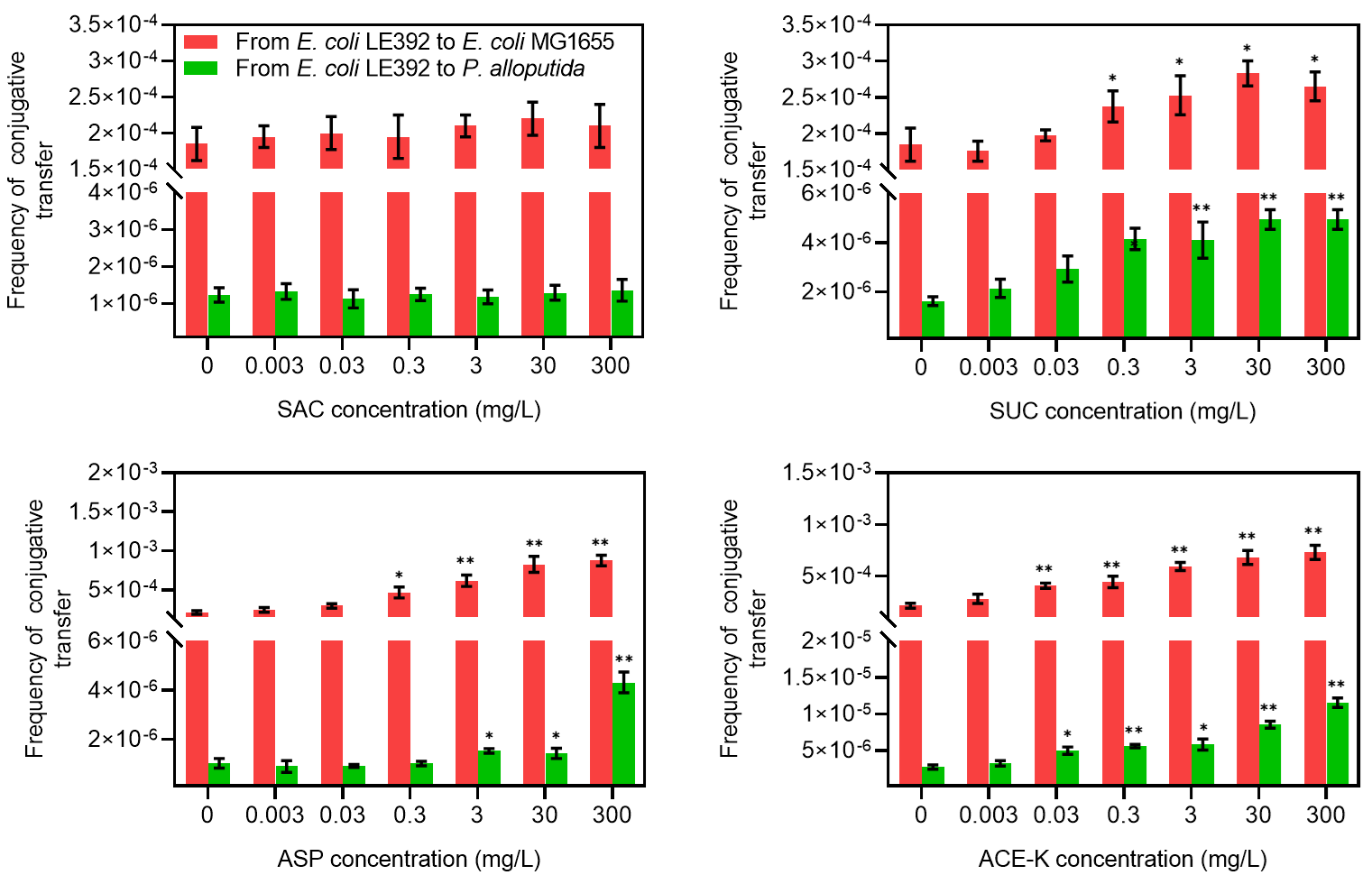


**Fig. S2.** Frequency of RP4 plasmid conjugative transfer (RP4 plasmid with Amp, Kan, Chl and Tet) from *E. coli* K-12 LE392 to *E. coli* K-12 MG1655 or to *P. alloputida* under exposure to four sweeteners (SAC, SUC, ASP and ACE-K). Significant differences between individual sweetener treated groups and the control were analysed using independent-sample t test and shown with * (*p* < 0.05) and ** (*p* < 0.01).


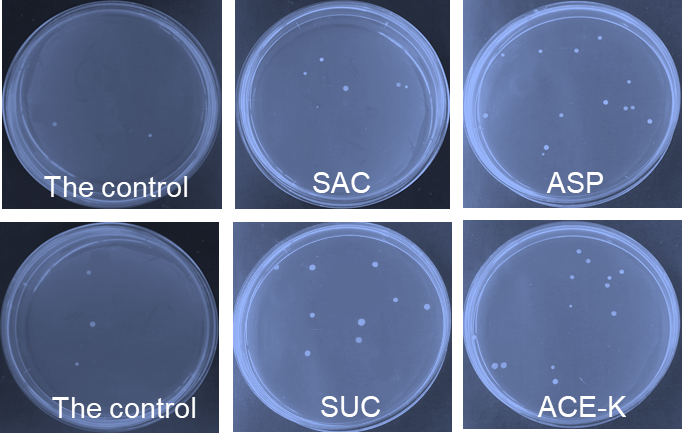


**Fig. S3.** Images of transconjugant number in selective plates after the mating system (donor *E. coli* K-12 LE392 and recipient *P. alloputida*) treated with or without 300 mg/L non-nutritive sweetener

**Fig. S4.** Linear regression modelling of fold changes in conjugative transfer frequency of pMS6198A plasmid


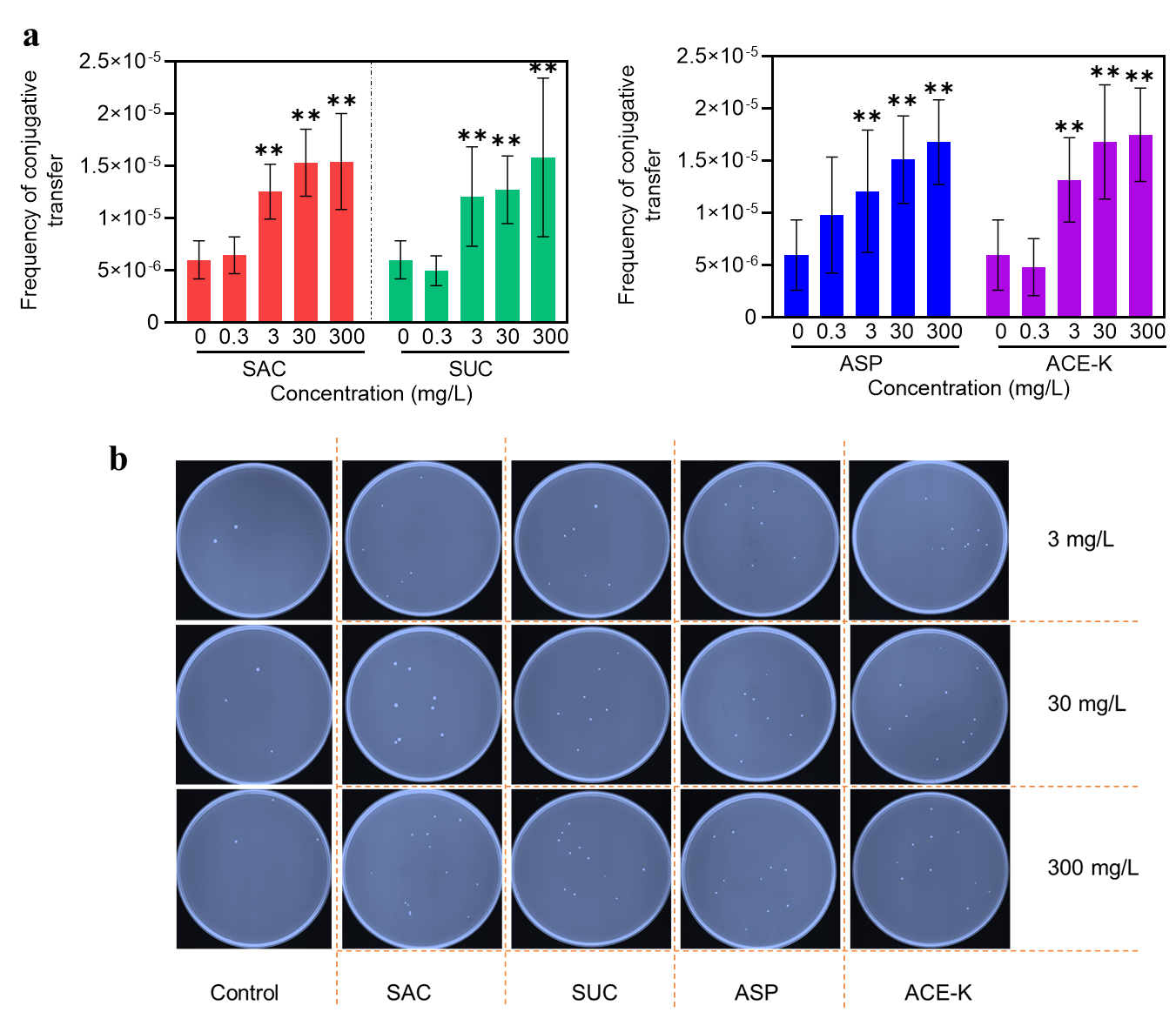


**Fig. S5.** (**a**) Frequency of pMS6198A plasmid conjugative transfer from *E. coli* K-12 MG1655 to *E. coli* J53 under exposure to four sweeteners (SAC, SUC, ASP and ACE-K). (**b**) Images of transconjugant number in selective plates after the mating system (donor *E. coli* K-12 MG1655 and recipient *E. coli* J53) treated with or without non-nutritive sweeteners


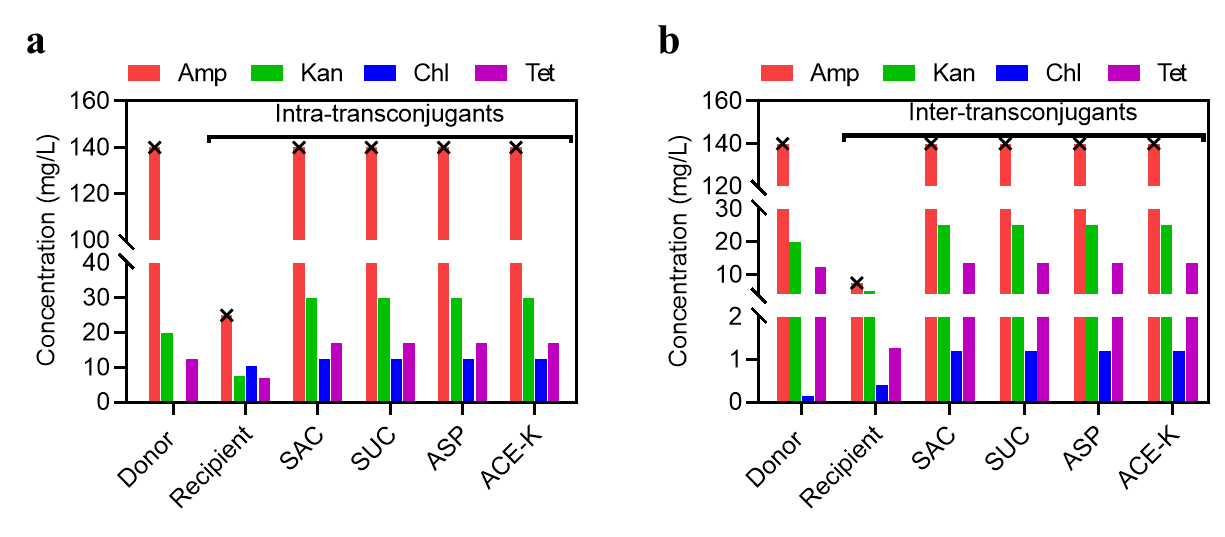


**Fig. S6.** MICs of donor, both recipients, intra- and intergenus transconjugants (induced by 3 mg/L non-nutritive sweeteners) toward antibiotics (Amp, Kan, Chl, and Tet), respectively (*N* = 3). The intra- and intergenus transconjugants had the resistance characteristics of both donor and recipient to the antibiotics. Those marked “×” indicates that the values are higher than their corresponding left Y axis values.


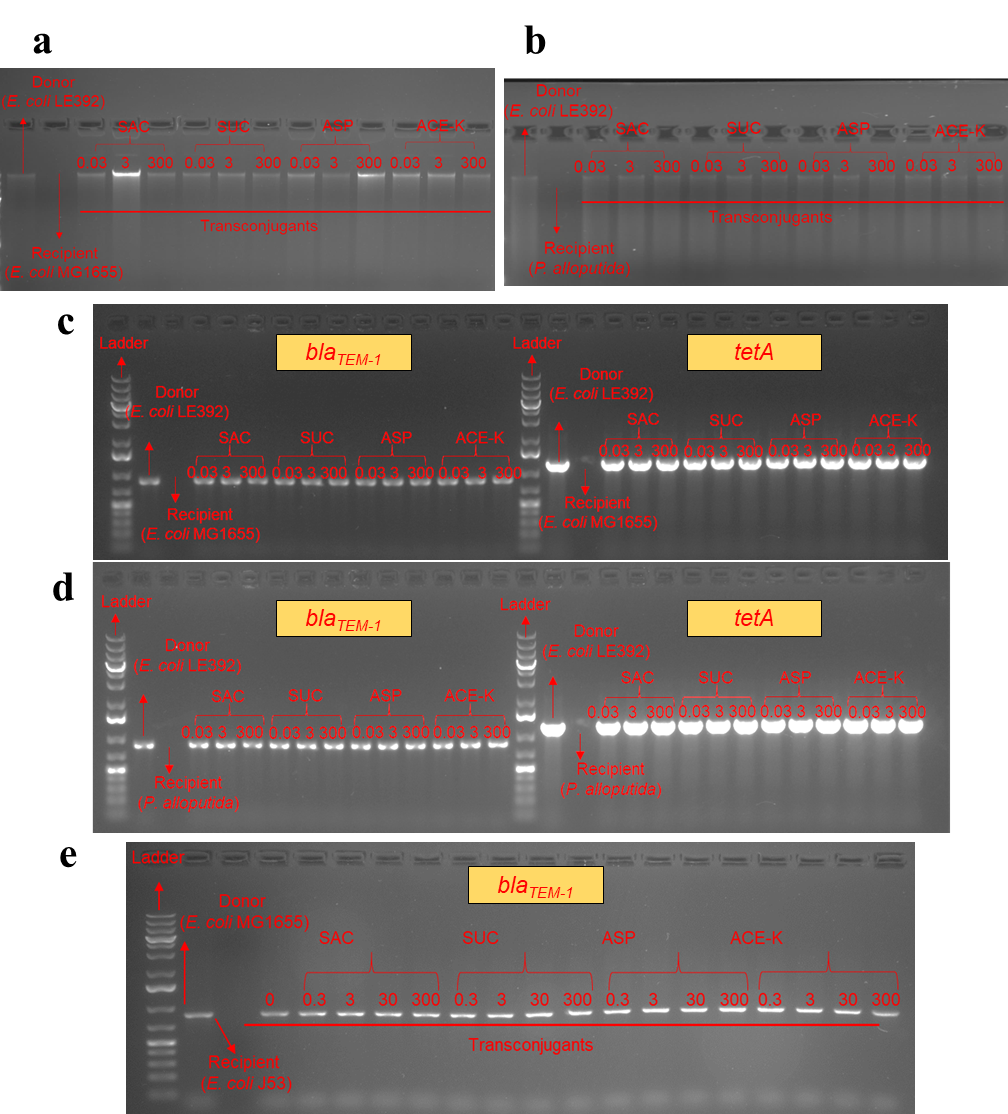


**Fig. S7.** Gel electrophoresis of (**a, b**) plasmid extraction and (**c, d, e**) PCR amplicons for ARGs (*bla_TEM-1_* and *tetA*) from donor (*E. coli* K-12 LE392), recipients (*E. coli* K-12 MG1655 and *P. alloputida*), and transconjugants. **a-d**, profiling of RP4 plasmid. **e**, PCR amplification of *bla_TEM-1_* from donor *E. coli* K-12 MG1655, recipient *E. coli* J53, and transconjugants.

**Fig. S8.** Linear regression modelling of fold changes in conjugative transfer frequency of RP4 plasmid (reverse conjugation)

**Fig. S9.** Frequency of RP4 plasmid conjugative transfer from transconjugant (*P. alloputida* containing RP4 plasmid) to *E. coli* K-12 MG1655 under exposure to four sweeteners (SAC, SUC, ASP and ACE-K)

**Fig. S10.** Percent of ROS production in the donor and two recipients after 2 h exposure to four sweeteners (SAC, SUC, ASP and ACE-K). Significant differences between individual sweetener treated groups and the control as well as no ROS scavenger addition and ROS scavenger addition were analysed using independent-sample t test and shown with * (*p* < 0.05) and ** (*p* < 0.01).


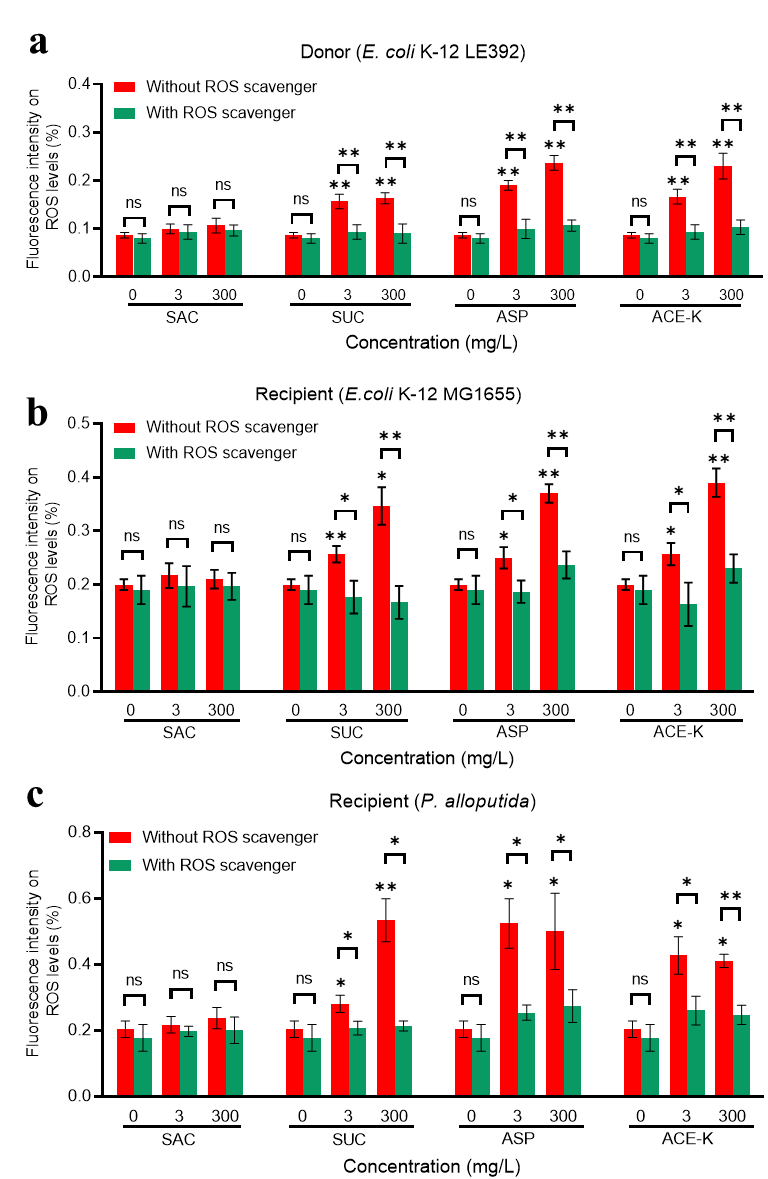


**Fig. S11.** Fluorescence intensity to indicate ROS levels (%) with or without ROS scavenger (thiourea) in the donor, recipient (*E. coli* K-12 MG1655), and recipient (*P. alloputida*).

**Fig. S12.** Percent of PI stained cells in the donor and two recipients after 2 h exposure to various concentrations of four non-nutritive sweeteners. Significant differences between individual sweetener treated groups and the control were analysed using independent-sample t test and shown with * (*p* < 0.05) and ** (*p* < 0.01).

**
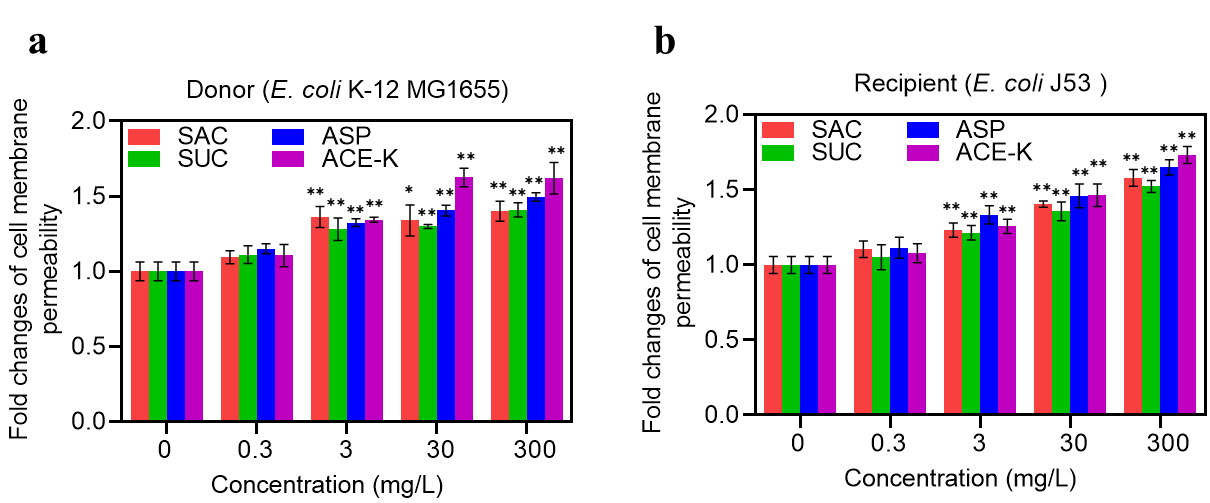
**

**Fig. S13.** Fold changes of cell membrane permeability in the (**a**) donor *E. coli* K-12 MG1655 and (**b**) recipient *E. coli* J53 after exposed to four non-nutritive sweeteners (*N* = 3).

**Fig. S14.** Influence of different pre-exposure of the donor (D) and two recipients (R) with SUC on the frequency of intra (a) and inter (b) conjugative transfer (*N* = 6). R+, means that the recipient was pre-exposed to 3 mg/L SUC for 2 h before conjugation experiment; D+, means that the donor was pre-exposed to 3 mg/L SUC for 2 h; SUC, means that the mixture of the donor and the recipient was directly exposed to 3 mg/L SUC for 8 h conjugation without pre-exposure. Significant differences between individual sweetener treated groups and the control were analysed using independent-sample t test and shown with * (*p* < 0.05) and ** (*p* < 0.01).

**Fig. S15.** Concentrations of four non-nutritive sweeteners in cell suspensions incubated by 8 h (*N* = 3). Initial concentrations of four sweeteners were selected at 3 mg/L. Significant differences between samples (the concentration was represented as c) from cell suspensions and the control (no cells; the concentration was represented as c_0_) were analysed using independent-sample t test and shown with ** (*p* < 0.01). No significant differences were observed (*p* > 0.05).

**Table S1. MICs (90%) of the donor and recipients toward non-nutritive sweeteners**

|  | **Non-nutritive Sweeteners, mg/L** | | | |
| --- | --- | --- | --- | --- |
| Cell | SAC | SUC | ASP | ACE-K |
| **Donor** |  |  |  |  |
| *E. coli* K-12 LE392 (RP4) | 1400 | > 1500 | > 1500 | > 1500 |
| *E. coli* K-12 MG1655 (pMS6198A) | > 3000 | > 3000 | > 3000 | > 3000 |
| **Recipient** |  |  |  |  |
| *E. coli* K-12 MG1655 | 1400 | > 1500 | > 1500 | > 1500 |
| *P. alloputida* | 1300 | > 1500 | > 1500 | > 1500 |
| *E. coli* J53 | > 3000 | > 3000 | > 3000 | > 3000 |

**Table S2. PCR primers used in this study**

| Primer | Amplicon size | Primer length | | Sequence | Annealing temperature |
| --- | --- | --- | --- | --- | --- |
| *bla_TEM-1_* Long FW | 861 bp | 24 bp | 5′-TTACCAATGCTTAATCAGTGAGGC-3′ | | 51.4℃ |
| *bla_TEM-1_*-Long RV |  | 25 bp | 5′-ATGAGTATTCAACATTTCCGTGTCG-3′ | |  |
| *tetA* Long FW | 1200 bp | 25 bp | 5′-CGTGTATGAAATCTAACAATGCGCT-3′ | | 51.9℃ |
| *tetA* Long RV |  | 19 bp | 5′-CCATTCAGGTCGAGGTGGC-3′ | |  |

**Table S3. Pearson’s linear correlation analysis of correlation between sweetener concentrations and fold changes in conjugative frequency**

|  | **Pearson’s linear correlation** | |
| --- | --- | --- |
|  | **Pearson correlation, *r*** | **Significance (2 tailed), *p*** |
| Conjugation within genera (RP4) | | |
| SAC | -0.36 | 0.43 |
| SUC | 0.76* | 0.047 |
| ASP | 0.98** | 0.00017 |
| ACE-K | 0.78* | 0.038 |
| Conjugation across genera (RP4) | | |
| SAC | -0.294 | 0.522 |
| SUC | 0.79* | 0.035 |
| ASP | 0.76* | 0.046 |
| ACE-K | 0.78* | 0.041 |
| Plasmid pMS6198A conjugation | | |
| SAC | 0.89* | 0.042 |
| SUC | 0.90* | 0.035 |
| ASP | 0.92* | 0.025 |
| ACE-K | 0.91* | 0.032 |
| Reverse conjugation (RP4) | | |
| SAC | 0.89 | 0.11 |
| SUC | 1.00** | 0.00025 |
| ASP | 0.99** | 0.010 |
| ACE-K | 0.96* | 0.042 |

Note: Correlations with *p* value (Sig. (2-tailed)) < 0.05 were considered as significant (*), *p* value < 0.01 were considered as very significant (**). A Pearson correlation coefficient (*r*) larger than 0.8 is highly positive linear correlation, *r* between 0.5 and 0.8 is moderately positive linear correlation.

**Table S4. mRNA genes relevant to ROS production in donor bacteria E. coli K-12 LE392 after treated by 3 mg/L of four sweeteners (SAC, SUC, ASP, ACE-K)**

| Gene | COG annotation | Fold change of gene expression^a^ | | | |
| --- | --- | --- | --- | --- | --- |
|  |  | **SAC** | **SUC** | **ASP** | **ACE-K** |
| *ahpC* | Alkyl hydroperoxide reductase subunit AhpC | 1.21 | 1.32 | 1.55* | 1.70** |
| *alkB* | Alkylated DNA repair dioxygenase AlkB | 1.21 | 2.30* | 2.51* | 1.00 |
| *gor* | Glutathione oxidoreductase | 1.15 | 0.99 | 1.21 | 1.40* |
| *katE* | Superoxide dismutase | 1.34 | 1.44* | 1.17 | 2.41** |
| *katG* | Superoxide dismutase | 1.03 | 0.99 | 1.15* | 1.09 |
| *sodA* | Superoxide dismutase | 1.21 | 1.27* | 1.79** | 1.76** |
| *sodB* | Superoxide dismutase | 1.22 | 1.20 | 1.21 | 1.52** |
| *sodC* | Cu/Zn superoxide dismutase | 1.38* | 1.73** | 1.50* | 2.84** |
| *tpx* | Lipid hydroperoxide peroxidase | 1.20 | 1.23 | 1.22 | 1.54** |
| *wrbA* | NAD(P)H:quinone oxidoreductase | 1.13 | 1.28* | 1.67** | 1.61** |
| *yfgD* | Putative oxidoreductase | 0.91 | 1.68* | 1.78* | 2.02* |
| *yghA* | Putative oxidoreductase | 1.29 | 1.71** | 1.29 | 2.95** |

^a^: Comparing with the control group without non-nutritive sweeteners treatment

*: *p* < 0.05, **: *p* < 0.01

**Table S5. mRNA genes relevant to the SOS response in donor bacteria E. coli K-12 LE392 after treated by 3 mg/L of four sweeteners (SAC, SUC, ASP, ACE-K)**

| Gene | COG annotation | Fold change of gene expression^a^ | | | |
| --- | --- | --- | --- | --- | --- |
|  |  | **SAC** | **SUC** | **ASP** | **ACE-K** |
| *dinB* | DNA polymerase IV~DNA polymerase IV%2C devoid of proofreading%2C damage-inducible protein P | 1.04 | 0.90 | 1.36* | 1.28 |
| *dps* | Stress-inducible DNA-binding protein | 1.55* | 1.85** | 1.95** | 3.04** |
| *phoP* | Two-component system DNA-binding transcriptional regulator | 1.27 | 1.52* | 1.83* | 1.73* |
| *sulA* | SOS cell division inhibitor | 1.38* | 1.32* | 1.48* | 1.63** |
| *umuC* | SOS mutagenesis and repair | 1.34 | 1.80* | 1.64* | 2.12* |
| *umuD* | SOS-response transcriptional repressor LexA | 1.52 | 1.02 | 1.73* | 1.25 |
| *uspA* | Universal stress global response regulator | 1.31 | 1.43* | 1.03 | 2.18** |
| *uspC* | Universal stress protein C | 1.34 | 2.73** | 0.72 | 0.94 |
| *uspD* | Universal stress protein D | 1.21 | 1.78* | 1.47* | 1.48* |
| *uspF* | Class II universal stress protein | 1.38 | 1.41* | 1.59** | 1.93** |
| *yebG* | DNA damage-inducible gene of the SOS regulon | 0.92 | 1.47* | 1.40* | 1.05 |

^a^: Comparing with the control group without non-nutritive sweeteners treatment

*: *p* < 0.05, **: *p* < 0.01

**Table S6. mRNA genes relevant to ROS production in recipient bacteria *P. alloputida* after treated by 3 mg/L of four sweeteners (SAC, SUC, ASP, ACE-K)**

| Gene | COG annotation | Fold change of gene expression^a^ | | | |
| --- | --- | --- | --- | --- | --- |
|  |  | **SAC** | **SUC** | **ASP** | **ACE-K** |
| *ahpC* | Alkyl hydroperoxide reductase subunit AhpC | 1.24 | 1.35* | 1.29* | 1.51** |
| *ahpF* | Alkyl hydroperoxide reductase subunit AhpF | 0.89 | 1.16 | 0.66** | 0.99 |
| *gor* | Glutathione oxidoreductase | 1.05 | 1.03 | 1.11* | 1.10* |
| *katE* | Superoxide dismutase | 1.04 | 1.06 | 1.28* | 1.17 |
| *katG* | Superoxide dismutase | 1.33* | 1.76** | 2.27** | 2.27** |
| *mqo* | Malate:quinone oxidoreductase | 1.19 | 1.56** | 1.44** | 1.82** |
| *sodA* | Superoxide dismutase | 0.98 | 1.16 | 1.76* | 1.53* |
| *sodB* | Superoxide dismutase | 1.27* | 1.41** | 1.28* | 1.38** |
| *tpx* | Arsenate reductase | 1.04 | 0.96 | 1.22* | 1.10 |
| *yfgD* | 2-Cys peroxiredoxin | 1.08 | 0.91 | 1.30* | 1.38* |
| *yhdF* | Oxidoreductase YhdF | 0.77 | 1.10 | 1.12 | 1.25** |

^a^: Comparing with the control group without non-nutritive sweeteners treatment

*: *p* < 0.05, **: *p* < 0.01

**Table S7. mRNA genes relevant to the SOS response in recipient bacteria *P. alloputida* after treated by 3 mg/L of four sweeteners (SAC, SUC, ASP, ACE-K)**

| Gene | COG annotation | Fold change of gene expression^a^ | | | |
| --- | --- | --- | --- | --- | --- |
|  |  | **SAC** | **SUC** | **ASP** | **ACE-K** |
| *dinB* | DNA polymerase IV~DNA polymerase IV%2C devoid of proofreading%2C damage-inducible protein P | 0.93 | 0.83 | 0.80 | 0.80 |
| *dps* | Stress-inducible DNA-binding protein | 1.10 | 0.95 | 1.26* | 1.27* |
| *phoP* | Two-component system DNA-binding transcriptional regulator | 1.23* | 1.29* | 1.57** | 1.70** |
| *sulA* | SOS cell division inhibitor | 1.08 | 1.21* | 0.98 | 1.19* |

^a^: Comparing with the control group without non-nutritive sweeteners treatment

*: *p* < 0.05, **: *p* < 0.01

**Table S8.** **Different pre-exposure** **of the donor and recipients with SUC before mating**

|  | Donor  (*E. coli* K-12 LE392) | | | Recipient  (*E. coli* K-12 MG1655) | | Recipient,  (*P. alloputida*) |
| --- | --- | --- | --- | --- | --- | --- |
| Treatment 1 | √ | |  | | |  |
| Treatment 2 | |  | | √ | |  |
| Treatment 3 | |  | | |  | √ |

Note: “√” means that 3 mg/L SUC was used in each pre-exposure group.

**Table S9. mRNA genes relevant to** **cell membrane in donor bacteria *E. coli* K-12 LE392 after treated by 3 mg/L of four sweeteners (SAC, SUC, ASP, ACE-K)**

| Gene | COG annotation | Fold change of gene expression^a^ | | | |
| --- | --- | --- | --- | --- | --- |
|  |  | **SAC** | **SUC** | **ASP** | **ACE-K** |
| *bamB* | Outer membrane protein assembly factor | 1.06 | 0.97 | 1.22* | 1.05 |
| *fecA* | Ferric citrate outer membrane transporter | 0.97 | 6.98** | 4.29** | 1.48* |
| *lamB* | Maltose outer membrane channel | 0.54 | 1.31* | 1.58* | 0.65 |
| *lolA* | Outer membrane protein A | 0.81 | 1.54* | 1.21 | 1.93** |
| *ompC* | Outer membrane porin protein C | 1.39* | 1.37* | 0.86 | 1.57** |
| *phoE* | Outer membrane phosphoporin protein E | 1.00 | 1.34* | '-Inf | 0.81 |
| *slp* | Outer membrane lipoprotein | 0.87 | 1.35* | 1.44* | 0.90 |
| *ybaY* | Outer membrane lipoprotein | 1.19 | 1.61** | 0.79 | 1.78** |
| *ybgE* | Putative inner membrane protein in cydABX-ybgE operon | 0.75 | 2.68** | '-Inf | 2.08** |
| *yfdY* | DUF2545 family putative inner membrane protein | 0.68 | 1.23* | 0.78 | 1.11 |
| *yihG* | Inner membrane acyltransferase | 0.82 | 1.80* | 0.89 | '-Inf |
| *yniB* | Putative inner membrane protein | 0.83 | 1.24* | 1.24* | 0.86 |
| *ynjI* | Inner membrane protein | 1.22 | 4.35* | 1.00 | 1.13 |
| *yohC* | Putative inner membrane protein | 1.11 | 1.31* | 1.24* | 1.49** |

^a^: Comparing with the control group without non-nutritive sweeteners treatment

*: *p* < 0.05, **: *p* < 0.01

**Table S10. mRNA genes relevant to cell membrane in recipient bacteria *P. alloputida* after treated by 3 mg/L of four sweeteners (SAC, SUC, ASP, ACE-K)**

| Gene | COG annotation | Fold change of gene expression^a^ | | | |
| --- | --- | --- | --- | --- | --- |
|  |  | **SAC** | **SUC** | **ASP** | **ACE-K** |
| *exbD* | TonB-gated outer membrane transporter gating inner membrane protein | 1.42 | 1.80* | 1.48 | 1.98** |
| *glmP* | Alginate biosynthesis membrane protein | 0.50 | 1.89* | 0.70 | 1.52* |
| *lolA* | Outer membrane lipoprotein carrier protein | 0.94 | 1.07 | 1.18* | 1.09 |
| *oprD* | Basic amino acid specific porin OprD | 1.27* | 1.40** | 1.24* | 1.53** |
| *oprF* | Porin F | 1.23* | 1.19 | 1.38* | 1.48** |
| *oprH* | Outer membrane protein H1 | 1.17 | 1.17 | 1.37* | 1.50** |
| *oprI* | Major outer membrane lipoprotein | 1.01 | 1.28* | 1.21* | 1.16* |
| *oprQ* | Outer-membrane porin D | 1.11* | 0.97 | 0.90 | 0.89 |
| *PP_0431* | Membrane protein | 1.16 | 1.07 | 1.45** | 1.47** |
| *PP_0523* | Membrane protein | 1.15 | 1.16 | 1.07 | 1.24* |
| *PP_0575* | Membrane protein | 0.94 | 1.13 | 1.26* | 1.50** |
| *PP_0647* | Membrane protein | 1.09 | 1.41* | 0.57 | 1.06 |
| *PP_0682* | Inner membrane protein | 1.04 | 1.05 | 1.50* | 1.29* |
| *PP_0717* | Membrane protein | 0.85 | 1.23* | 0.85 | 0.95 |
| *PP_1029* | Membrane protein | 1.01 | 1.20* | 0.88 | 1.09 |
| *PP_1193* | Membrane protein | 1.94** | 2.32** | 2.00** | 2.75** |
| *PP_1460* | Membrane protein | 1.18* | 1.20* | 1.06 | 1.11 |
| *PP_2104* | Membrane protein | 0.85 | 1.02 | 1.16* | 0.96 |
| *PP_2506* | Membrane protein | 1.55* | 1.38* | 1.04 | 1.19 |
| *PP_2684* | Membrane protein | 1.71* | 1.59* | 1.07 | 1.85* |
| *PP_2721* | Membrane protein | 1.23* | 1.26* | 0.51 | 1.23* |
| *PP_2726* | Membrane protein | 1.06 | 1.16* | 0.97 | 1.15* |
| *PP_2733* | Membrane protein | 1.00 | 1.16 | 1.72* | 1.90* |
| *PP_2915* | Membrane protein | 1.04 | 1.36* | 1.30* | 0.95 |
| *PP_3169* | Membrane protein | 1.03 | 1.06 | 1.20 | 1.24* |
| *PP_3389* | Membrane protein | 0.96 | 1.25* | 0.79 | 0.73 |
| *PP_3579* | Membrane protein | 1.12 | 1.08 | 1.47* | 1.04 |
| *PP_4057* | Membrane protein | 1.23* | 1.14 | 1.14 | 1.31* |
| *PP_4118* | Membrane protein | 0.85 | 1.188 | 1.05 | 0.97 |
| *PP_4272* | Membrane protein | 1.10 | 0.58 | 1.15* | 1.15* |
| *PP_4289* | Membrane protein | 1.01 | 1.40** | 1.06 | 1.52** |
| *PP_4465* | Porin | 1.00 | 1.19* | 1.11 | 1.04 |
| *PP_4825* | MarC family membrane protein | 1.07 | 1.37* | 0.87 | 1.13 |
| *PP_4850* | Membrane protein | 0.83 | 1.36* | 0.82 | 0.98 |
| *PP_5091* | Membrane protein | 1.07 | 1.08 | 1.01 | 1.43** |
| *PP_5334* | Membrane protein | 1.22* | 1.19 | 1.36* | 1.13 |
| *PP_5423* | Membrane protein | 1.02 | 1.26* | 0.14 | 0.30 |
| *PP_5460* | Membrane protein | 1.07 | 1.11 | 1.03 | 1.17* |
| *PP_5505* | Transmembrane protein | 1.87* | 1.33* | 0.76 | 1.31 |
| *PP_5535* | Membrane protein | 1.54* | 1.87** | 2.90** | 2.84** |
| *PP_5563* | Membrane protein | 1.23* | 1.13 | 0.69 | 0.98 |
| *PP_5718* | Membrane protein | 1.64* | 1.40 | 0.85 | 1.35 |
| *yohC* | Inner membrane protein | 1.23 | 1.33* | 1.06 | 1.63** |
| *yphA* | Inner membrane protein | 1.25 | 1.38* | 1.28 | 1.62** |

^a^: Comparing with the control group without non-nutritive sweeteners treatment

*: *p* < 0.05, **: *p* < 0.01

**Table S11. Genes relevant to the relaxosome formation, mating pair formation (Mpf), DNA replication and pilus channel formation in RP4 plasmid after exposure to four sweeteners**

| Gene | Gene description | Fold change of gene expression^a^ | | | |
| --- | --- | --- | --- | --- | --- |
|  |  | **SAC** | **SUC** | **ASP** | **ACE-K** |
| *korC* | Regulation of genes expression | 0.66* | 0.75* | 0.44** | 0.43** |
| *traG* | Conjugative transfer: assembly and aggregate stability | 1.25 | 1.34 | 1.34 | 1.76** |
| *traC1* | DNA primase and ssDNA binding protein | 1.35 | 1.46** | 1.72** | 2.34** |
| *traC2* | DNA primase and ssDNA binding protein | 0.37 | 2.04* | 2.00 | 0.84 |
| *traA* | conjugal transfer protein | 1.11 | 1.07 | 1.70** | 1.56** |
| *traB* | P-type conjugative transfer protein | 1.20 | 1.52** | 1.38* | 2.10** |
| *traF* | Conjugal pilus assembly protein TraF | 0.96 | 1.17 | 1.30* | 1.69* |
| *traP* | Type II secretion system prepilin peptidase | 1.72* | 1.38 | 2.60** | 3.00** |

^a^: Comparing with the control group without non-nutritive sweeteners treatment

*: *p* < 0.05, **: *p* < 0.01

**Table S12. Fold changes of expression of core genes related to the adhesive pilus in donor cell after exposure to four sweeteners**

| Gene | Gene description | Fold change of gene expression^a^ | | | |
| --- | --- | --- | --- | --- | --- |
|  |  | **SAC** | **SUC** | **ASP** | **ACE-K** |
| *ecpA* | ECP pilin | 2.07* | 4.20* | 1.82* | 2.08* |
| *fimH* | Minor component of type 1 fimbriae | 1.50* | 1.97* | 1.24 | 1.65* |
| *yadV* | Putative periplasmic pilin chaperone | 0 | Inf | Inf | Inf |
| *ybgP* | Putative fimbrial chaperone YbgP | '-Inf | 1.33 | 2.78** | 1.75 |
| *ynfD* | DUF1161 family periplasmic protein | 1.15 | 1.43* | 1.24 | 2.22** |

^a^: Comparing with the control group without non-nutritive sweeteners treatment

*: *p* < 0.05, **: *p* < 0.01

**Movie S1.**

Microfluidic time-lapse imaging showing *gfp*-RP4 plasmid spontaneous conjugation between donor cells (red fluorescence) and recipient cells (no fluorescence). Plasmid acquisition in transconjugant cells is confirmed by the formation of only green fluorescence, which corresponds to *gfp* (on the plasmid) expression in the recipient (becoming transconjugant). Merge of phase contrast, red and green fluorescence channels are shown in the panel. Cells were suspended in PBS with 100 *µ*M IPTG at 30℃. Scale and time are indicated.

**Movie S2.**

Microfluidic time-lapse imaging showing the conjugative transfer of *gfp*-RP4 plasmid between donor cells (red fluorescence) and recipient cells (no fluorescence) under exposure to a non-nutritive sweetener ACE-K (300 mg/L). Plasmid acquisition in transconjugant cells is confirmed by the formation of only green fluorescence, which corresponds to *gfp* (on the plasmid) expression in the recipient. Merge of phase contrast, red and green fluorescence channels are shown and there is a succession of 20 conjugation events in the movie. Cells were suspended in PBS with 100 *µ*M IPTG at 30℃. Scale bar is 1 *µ*m and time is indicated.

**References**

1. Nolivos S, Cayron J, Dedieu A, Page A, Delolme F, Lesterlin C. Role of AcrAB-TolC multidrug efflux pump in drug-resistance acquisition by plasmid transfer. Science. 2019;364(6442):778-82.

2. Subedi B, Kannan K. Fate of non-nutritive sweeteners in wastewater treatment plants in New York State, U.S.A. Environ Sci Technol. 2014;48(23):13668-74.

3. Subedi B, Lee S, Moon HB, Kannan K. Emission of non-nutritive sweeteners, select pharmaceuticals, and personal care products through sewage sludge from wastewater treatment plants in Korea. Environ Int. 2014;68:33-40.
